# Aberrant gene expression by Sertoli cells in infertile men with Sertoli cell-only syndrome

**DOI:** 10.1371/journal.pone.0216586

**Published:** 2019-05-09

**Authors:** Darius A. Paduch, Stephanie Hilz, Andrew Grimson, Peter N. Schlegel, Anne E. Jedlicka, William W. Wright

**Affiliations:** 1 Department of Urology, Weill Cornell Medical College, New York, NY, United States of America; 2 Department of Molecular Biology and Genetics, Cornell University, Ithaca, NY, United States of America; 3 Department of Neurological Surgery, University of California, San Francisco, California, United States of America; 4 Genomic Analysis and Sequencing Core Facility, Johns Hopkins Bloomberg School of Public Health, Baltimore, Maryland, United States of America; 5 Department of Biochemistry and Molecular Biology, Johns Hopkins Bloomberg School of Public Health, Baltimore, Maryland, United States of America; 6 Consulting Research Services, Inc, North Bergen, N.J., United States of America; University Hospital of Münster, GERMANY

## Abstract

Sertoli cell-only (SCO) syndrome is a severe form of human male infertility seemingly characterized by the lack all spermatogenic cells. However, tubules of some SCO testes contain small patches of active spermatogenesis and thus spermatogonial stem cells. We hypothesized that these stem cells cannot replicate and seed spermatogenesis in barren areas of tubule because as-of-yet unrecognized deficits in Sertoli cell gene expression disable most stem cell niches. Performing the first thorough comparison of the transcriptomes of human testes exhibiting complete spermatogenesis with the transcriptomes of testes with SCO syndrome, we defined transcripts that are *both* predominantly expressed by Sertoli cells and expressed at aberrant levels in SCO testes. Some of these transcripts encode proteins required for the proper assembly of adherent and gap junctions at sites of contact with other cells, including spermatogonial stem cells (SSCs). Other transcripts encode GDNF, FGF8 and BMP4, known regulators of mouse SSCs. Thus, most SCO Sertoli cells can neither organize junctions at normal sites of cell-cell contact nor stimulate SSCs with adequate levels of growth factors. We propose that the critical deficits in Sertoli cell gene expression we have identified contribute to the inability of spermatogonial stem cells within small patches of spermatogenesis in some SCO testes to seed spermatogenesis to adjacent areas of tubule that are barren of spermatogenesis. Furthermore, we predict that one or more of these deficits in gene expression are primary causes of human SCO syndrome.

## Introduction

Infertility is a problem that besets approximately 15% of couples of childbearing age, with men being the sole cause of the couple’s infertility approximately one-third of the time [1]. A severe form of human male infertility is characterized histologically by the apparent lack of all spermatogenic cells in almost all seminiferous tubules, a condition called Sertoli cell-only (SCO) syndrome. While tubules without germ cells are markedly atrophied, small segments of seminiferous tubules within some SCO testes are dilated, exhibit active spermatogenesis and produce sperm [2]. As the cellular foundation of spermatogenesis is the spermatogonial stem cell (SSC), the question arises as to why self-renewing replication of the SSCs in these segments does not generate new stem cells that can colonize adjacent germ cell-deficient areas of tubule and thereby seed spermatogenesis. Normally, SSCs have this capacity [3, 4]. We propose that one of the reasons that this does not occur is that the somatic cells associated with those barren areas of tubule do not form a functional stem cell niche. Thus, identifying deficits in gene expression by those somatic cells may provide new insights into the mechanistic basis for this form of male infertility and serve as a potential first step toward developing new therapies for men whose apparently SCO testes contain some SSCs. This study focuses primarily on Sertoli cells, the only somatic cells in direct physical contact with SSCs, and an important source of growth factors that regulate the replication and differentiation of SSCs and their immediate progeny, transit amplifying progenitor spermatogonia [5, 6]. Thus, Sertoli cells within a fertile testis are both the architects of the stem cell niche and the source of important growth factors that act within it. It follows that aberrant gene expression by Sertoli cells may disable some stem cell niches, and, thus, be one cause of Sertoli cell-only syndrome.

The analyses presented herein are an important step towards the full characterization of aberrant gene expression by Sertoli cells within human SCO testes. We collected testicular biopsies from patients who were undergoing micro-testicular sperm extraction, and then performed the first thorough comparison of the transcriptomes of testis with complete spermatogenesis (i.e. spermatogenesis was either quantitatively or qualitatively normal) with the transcriptomes of testes with SCO syndrome. We then focused on transcripts expressed primarily by Sertoli cells in a fertile human testis. We refer to these as Sertoli cell signature transcripts.

Because the transcriptome of normal human Sertoli cells has yet to be analyzed directly, we used a two-step bioinformatic approach to define human Sertoli cell signature transcripts. This approach was founded on the following considerations. First, Sertoli cells of all mammalian species fulfill the same essential functions, which are determined in large part by their transcriptomes. Second, the transcriptomes of adult rat Sertoli cells and other testicular cell types are known and thus, rat Sertoli cell signatures transcripts can be identified directly [7, 8]. Third, that rat genes and their human orthologues predominantly encode proteins with the same fundamental functions. Thus, we anticipated that the compendium of rat and human Sertoli cell signature transcripts would be largely congruent. Fourth, the transcriptomes of a human Leydig and spermatogenic cells and numbers of these cells in an adult testis have been defined [9–12]. Thus, Leydig and spermatogenic cell transcriptomes could be used to estimate the contribution of each cell type to the overall testis content of a given transcript, thereby allowing us to winnow and refine our list of human Sertoli cell signature transcripts.

A goal of our studies was to identify signature transcripts whose aberrant expression might explain important characteristics of human SCO testes and their dysfunctional niches. The first characteristic is that the SCO Sertoli cells fail to form highly organized and dynamic junctions with each other and with spermatogenic cells [13, 14]. In a fertile testis, junctions between Sertoli cells create the blood-testis barrier and thus the intratubular environment required by spermatogenesis [15]. Sertoli cells also form junctions with SSCs and other spermatogenic cells, and these junctions provide the positional information needed for the stem cells to undergo the asymmetric divisions that produce one new stem cell and one progenitor spermatogonium [16, 17]. The disorganization of cell-cell junctions in SCO testes implies that such positional information is diminished.

A second characteristic of SCO testes is the inability of its few SSCs to expand in number, fill empty niches and seed new areas of tubule with spermatogenesis. We hypothesized that one reason that SSC expansion does not occur is inadequate production of essential paracrine regulators of these stem cells by Sertoli cells. Mouse Sertoli cells produce a number of such factors, including glial cell line-derived neurotrophic factor (GDNF), which stimulate replication and migration of these stem cells and suppresses their differentiation [6, 18]. We previously demonstrated that Sertoli cells in human SCO testes produce markedly less GDNF than their counterparts in fertile testes [19]. However, as multiple paracrine factors act in concert to regulate SSCs, we sought to determine if Sertoli cells in human SCO testes mis-express other important paracrine regulators of these stem cells.

Our analyses provide the first detailed comparison of the transcriptomes of human testes with complete spermatogenesis to transcriptomes of testes with SCO syndrome and identify deficits in gene expression by Sertoli cells that potentially explain the two characteristics of the human SCO testis noted above. Importantly, these explanations are strengthened by published studies on rodents that document the importance of specific genes to the functions of niches and the SSCs that they shelter.

## Materials and methods

### Human subjects

Testis biopsies were obtained from 11 patients, who were undergoing micro-testicular sperm extraction as part of treatment for infertility [2, 20]. All patients gave written informed consent for the collection of the biopsies, and The Institutional Review Board of Weill Medical College of Cornell University approved both the collection of tissues and their analyses. Table 1 describes the age, weight and height of each patient as well as their peripheral blood cytogenetics (determination of karyotype and a screen for Y chromosome microdeletions), success of sperm retrieval and fertilization by ICSI, serum FSH levels, and testicular histology. Each biopsy was separated into two pieces; the first was used for histological analysis; the second (~15–40 mg) was immediately snap frozen in liquid nitrogen and subsequently used for transcriptional analysis [20]. Tissue for histological analysis was fixed in 4% buffered paraformaldehyde, embedded in paraffin and 5-micron hematoxylin and eosin-stained stained histological sections prepared. Three sections were obtained from three different levels of each biopsy. Dr. Paduch and a member of the Weill-Cornell Department of Pathology independently evaluated the histology of each biopsy, which they characterized as: *normal*, no substantial abnormalities; exhibiting mild or moderate *hypospermatogenesis*, spermatogenesis is qualitatively normal, but there are reduced numbers of germ cells in tubule cross-sections; *maturation arrest*, a failure of spermatogenesis during meiosis or spermiogenesis; or *SCO*, exhibiting Sertoli cell-only syndrome. S1 Fig. shows representative micrographs of a normal testis and of testes exhibiting either mild or moderate hypospermatogenesis. We have previously published a representative photomicrograph of a section of an SCO testis, which appears devoid of all spermatogenic cells [19]. In our current study, data from testes with normal spermatogenesis or with hypospermatogenesis are combined under a single category, complete spermatogenesis (CS). When there was significant variation in the histology of tubules within a biopsy, we estimated the percentages of tubules exhibiting each of the four characteristics described above. Dr. Paduch and the pathologist concurred on the histology of the 11 testis biopsies that are the subject of this study.

**Table 1:** Description of donors.

Sample	Age	Weight	Height	Sperm	Eggs	Embryos**	FSH (mIU)	Cytogenetic
	(yrs.)	(lbs.)		Retrieved*	Injected (ICSI)		/ml serum	Analysis
**C1**	32	176	5'7"	Yes	13	7	5.4	Normal
**C2**	55	278	5'7"	Yes	4	1	8.9	Normal
**C3**	33	259	6'6"	Yes	14	6	10.03	Normal
**C4**	31	181	5'9"	Yes	14	8	No data	Normal
**Mean**	**37.8**	**223.5**	**5'10"**	** **	**11.25**	**5.5**	**8.11**	
**SCO5**	30	185	6'0"	Yes	13	9	15	Normal
**SCO7**	24	174	5'5"	Yes	13	5	31.87	Normal
**SCO8**	34	155	5'6"	Yes	20	16	15.2	Normal
**SCO9**	42	304	6'0"	Yes	19	1	8.1	Normal
**SCI10**	35	248	6'2"	Yes	8	1	22.42	Normal
**SCO11**	25	240	6'1"	Yes	11	3	15.39	Kleinfelter
**SCO12**	36	136	5'7"	Yes	8	6	29	Normal
**Mean**	**32.3**	**206**	**5'10"**	** **	**13**	**6**	**19.57**	** **

* Retrieval during micro-TESE

**Developed to at least 4-cell stage

### Transcriptional analysis of testis biopsies

Total RNA was isolated from frozen biopsies by use of the miRCURY RNA Isolation Kit (EXIQON, Wolbern, MA). Frozen tissue was homogenized in lysis buffer with TissueRuptor (Quigen, Valencia, CA), and total RNA isolated following manufacturer’s instructions. RNA purity and integrity were evaluated with an Agilent Bioanalyzer 2100 (Agilent Technologies, Lexington, MA).

Non-stranded RNAseq libraries were prepared (TruSeq, Illumina; 15 cycles of amplification) and sequenced on an Illumina HiSeq 2500 (50 bp; single read; average library depth was 17 million reads). Reads were aligned to genes and numbers of transcripts quantified using the Burrows-Wheller Alignment tool and HTSeq [21, 22]. The Pre-normalized data were filtered using a threshold of > 0.4 CPMs (counts per million mapped reads) in at least 2 of the libraries prepared from testes with complete spermatogenesis (https://support.bioconductor.org/p/60205/) and purged of genes that did not encode mRNAs. edgeR [23] was used to calculate normalized gene expression from raw counts of transcripts for human genes (iGenomes hg19 gene annotation file). CPM values for each library then were normalized, log2 transformed, and significant differences in expression between the two sets of testes defined as a false discovery rate (FDR) < 0.05.

To confirm that the threshold of 0.4 CPM noted above was a good choice for a cutoff in our whole transcriptome differential expression analysis, we examined the relationship between transcript expression and logFC (MA plot), as well as FDR p-value (volcano plot), and observed that there was no over-abundance of low-expression genes with apparent outlier logFC values that were being identified as differentially expressed. R script used for analysis of the sequencing data is available online (https://github.com/SRHilz/SCO).

As somatic cells are enriched in SCO testes, we initially evaluated expression of well-characterized transcripts after data were normalized for beta actin mRNA. Two tailed T-tests were used to compare beta-actin-normalized CPM values in CS and SCO testes. These comparisons were performed with GraphPad Prism 6.0 (La Jolla, CA) and significant differences defined as p≤0.005.

### Defining signature transcripts of human Sertoli cells

The very limited size of the testis biopsies precluded isolating Sertoli cells prior to sequencing. To circumvent this limitation, we first assessed the entire testis transcriptome of each sample, and then focused our analyses on transcripts that were predominantly expressed by human Sertoli cells and thus were signature transcripts for this cell-type. We developed a two-step screen to define such transcripts, which is described in detail S1 Text. Supplementary Methods. The first step was based on both the knowledge that Sertoli cells of all mammalian species fulfill the same essential functions and on our expectation that orthologous genes encode analogous functions. Thus, we used the published transcriptomes of rat Sertoli cells, Leydig cells, spermatogonia, pachytene spermatocytes and round spermatids to identify transcripts that were expressed at least 4-fold higher by rat Sertoli cells than by any of the other 4 cell types [8, 24]. We then identified orthologues of these transcripts in human testes.

The second step estimated the level of expression of orthologous transcripts by human Sertoli cells and identified those transcripts that met the criteria for signature transcripts (described below). To generate this estimate, we combined the human testis transcriptome defined by our analyses with published transcriptomes of human Leydig cells and spermatogenic cells and the relative numbers of each cell type, including Sertoli cells, in a human testis [9–12]. We calculated the contribution of Leydig cells and of each germ cell type to the amount of each transcript in the total testis transcriptome. Then we estimated the expression of a transcript by Sertoli cells with complete spermatogenesis by subtracting the total amount of a given transcript expressed by Leydig cells plus spermatogenic cells from the total testis transcriptome. We defined signature transcripts of Sertoli as meeting the following criteria: (a) Sertoli cells are the source of at least 60% of that transcript in the testis, (b) the expression of the transcript by Sertoli cells was at least twice that of Leydig cells, and (c) expression by Sertoli cells was greater than expression by any germ cell. Prior to comparing expression of Sertoli cells signature transcripts in testes with complete spermatogenesis with testes with SCO syndrome, CPM values for the signature transcripts in each sample were normalized to the same median value and data statistically reanalyzed as described above for the total testis transcriptomes

Finally, as signature transcripts are essential to Sertoli cell differentiated function and as these functions are important to fertility of all male mammals, we tested the prediction that most human Sertoli cell signature transcripts were also expressed by mature mouse Sertoli cells, which have been thoroughly studied by single-cell RNA sequencing [25]. To this end, we searched for orthologues of the human Sertoli cell signature transcripts in the mature mouse Sertoli cell transcriptome.

### Identification of FGF8 as a product of human Sertoli cells and measurement of FGF8 mRNA by real time PCR

Our analyses identified FGF8 as a product of Sertoli cells that was expressed at remarkably reduced levels in the SCO testis. As FGF8 is a regulator of mouse SSCs [26] we used FACs analysis, to confirm that this growth factor is expressed primarily by Sertoli cells in the human testis, and real time RT-PCR, to corroborate that FGF8 mRNA expression is marked reduced in SCO testes. Details of these two procedures are described in Supplemental methods.

## Results

### Comparison of the transcriptomes of testes with complete spermatogenesis with the transcriptomes of testes with SCO syndrome

Table 1 describes the donors of the biopsies, and Table 2 describes the histology of the biopsies. Testes from these patients were assigned to one of two groups based on histology. Spermatogenesis was complete in four of the biopsies (C1 to C4). Sertoli cell-only syndrome was evident in all tubules of 6 of the seven other testes. The histology of the seventh SCO testis, SCO11, was mixed. Eighty percent of the tubules were SCO and 20% exhibited severe hypospermatogenesis. The patients in the two groups were similar in age, weight and height, but patients with SCO syndrome had elevated serum FSH levels. All patients but one had a normal karyotype and a normal Y chromosome. The donor of sample SCO11 was diagnosed with Kleinfelter syndrome. Sperm were retrieved from one or more segments of dilated tubules and fertilization by intracytoplasmic sperm injection was achieved for all patients. Dilated tubule segments in testes with SCO syndrome could not be collected for RNA seq analysis, since all were required for sperm retrieval and subsequent ICSI.

**Table 2:** Histology of the biopsies obtained from donors.

Sample	Diagnosis from	Description of Tubules in Biopsy
	Biopsy	
**C1**	Normal Spermatogenesis	Normal spermatogenesis in 100% of tubules.
**C2**	Normal Spermatogenesis	Normal spermatogenesis in 95% of tubules.
**C3**	Hypospermatogenesis	Mild hypospermatogenesis; focal germ cell degeneration
**C4**	Hypospermatogenesis	Moderate hypospermatogenesis: 50%; Maturation arrest: 15%; SCO:35%.
**SCO5**	Sertoli cell-only	SCO 100%
**SCO7**	Sertoli cell-only	SCO 100%
**SCO8**	Sertoli cell-only	SCO 100%
**SCO9**	Sertoli cell-only	SCO 100%
**SCI10**	Sertoli cell-only	SCO 100%
**SCO11**	Sertoli cell-only	SCO: 80%; severe hypospermatogenesis: 20%
**SCO12**	Sertoli cell-only	SCO 100%

**Fig 1:**
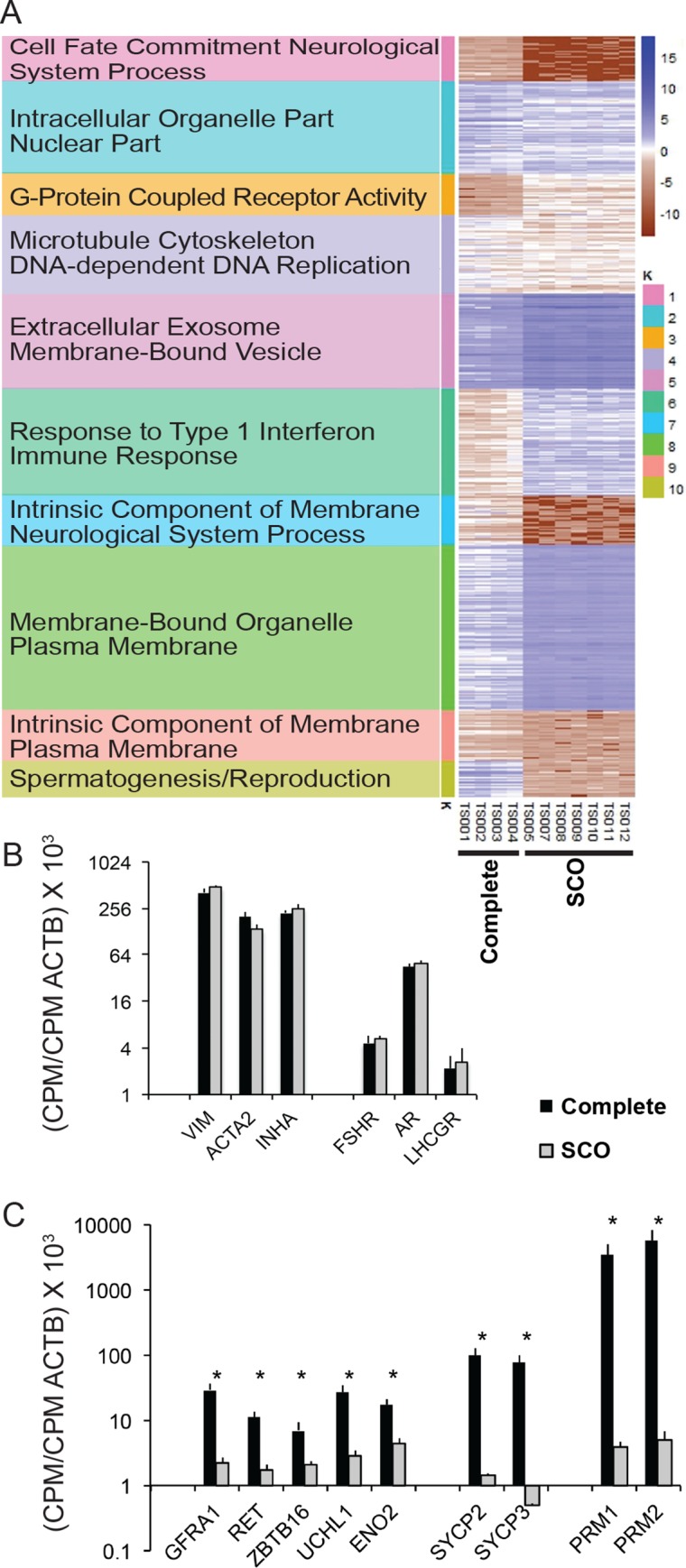
Comparison of the transcriptomes of testes with complete spermatogenesis with the transcriptomes of testes with SCO syndrome. (A) K means cluster analysis comparing the transcriptomes of testes with complete spermatogenesis with testes with SCO syndrome. Genes were clustered under 5 Gene Ontology terms; one or two representative terms per cluster are listed here. (B) The abundance in of a few well-characterized transcripts considered markers of three testicular somatic cells: Sertoli cells (VIM), peritubular myoid cells (ACTA2), and Leydig cells (INHA). Also shown are transcripts encoding receptors for three hormones required for normal testis function: FSHR, AR, and LHCGR. (C) Abundance of transcripts expressed by human SSCs (GFRA1, RET, ZBTB16, UCHL1, ENO2), by spermatocytes (SYCP2, SYCP3) and by round spermatids (PRM1, PRM2). Data in panels B and C are presented as CPM values in the total testis transcriptome divided by the CPM value for beta actin in the same sample. Asterisks over a pair of bars identify significant differences between testes with complete spermatogenesis and SCO testes (p<0.005).

RNA was isolated from each biopsy and the transcriptomes determined by RNAseq. These data have been uploaded to the NCBI database of Genotypes and Phenotypes (Study Name: NICHD/SCO_Syndrome; Accession Number phs001777.vi.p1). A total of 17,208 distinct mRNAs were identified in testes, and of those 4,748 and 5,274 transcripts had significantly higher or lower expression, respectively, in SCO testes. These differentially expressed transcripts sorted into 10 clusters based on expression levels and subsequent Gene Ontology (GO) enrichment analysis (Fig 1A). As anticipated, transcripts with GO terms “Spermatogenesis”.and “Reproduction” were substantially less abundant in SCO testes. However, genes with GO terms “G-protein coupled receptor activity” were enriched in the transcriptomes of SCO testes. A likely explanation for this enrichment was that the somatic cells in the testis express many G-protein coupled receptors and somatic cells are significantly enriched in the SCO testis. In fact, many transcripts expressed primarily by somatic cells, including Sertoli cells, were enriched approximately 3-fold in SCO testes; so, too, was beta actin mRNA. Therefore, to initially compare expression of characterized- somatic cell and germ cell transcripts in the two patient populations, we normalized CPMs (counts per million map reads) for each transcript to CPMs of beta actin mRNA. Fig 1B shows data for vimentin (VIM), smooth muscle actin (ACTA2) and inhibin alpha (INHA), which are considered markers of Sertoli cells, peritubular myoid cells and Leydig cells, respectively [19, 27, 28]. Normalized expressions of these three transcripts were similar in human testes with complete spermatogenesis and in testes exhibiting SCO syndrome. Testes in both groups also expressed similar levels of transcripts that encode receptors for the three hormones essential to male fertility, FSH, testosterone and LH (Fig 1B). Thus, some genes essential for male fertility are expressed normally by somatic cells in SCO testes.

We previously reported that the expression of a marker of human SSCs, GFRA1 mRNA, was significantly reduced to 10% of its expression in testes with complete spermatogenesis, while expression of a marker of spermatocytes and spermatids, DDX4 mRNA was reduced to 0.1% [19]. To confirm and extend those results we compared beta actin-normalized expression of 9 different germ cell transcripts in the two groups of testes (Fig 1C). Consistent with results of the study noted above, expression of GFRA1 mRNA in SCO testes was 8% of its expression in testes with complete spermatogenesis. Expressions of four other markers of SSCs (Ret, ZBTB16, UCLH1 and ENO2) were also reduced to 19% to 30% of the levels in testes with complete spermatogenesis. Transcripts expressed by spermatocytes (SYCP2 and SYCP3) or by spermatids (PRM1 and PRM2) were reduced to an even greater extent, approximately 1% and 0.1%, respectively, of what was measured in testes with complete spermatogenesis. The concentrations of germ cell-expressed transcripts in individual biopsies were consistent with their histology (S2 Fig). All of these transcripts were markedly higher in biopsies with complete spermatogenesis than in those exhibiting SCO syndrome. Furthermore, the concentrations of these transcripts were higher in testes with normal spermatogenesis (C1, C2) than in testes with hypospermatogenesis (C3, C4) (S2 Fig). Finally, our analyses demonstrated that in SCO testes there is a much greater decrease in the concentrations of the four transcripts expressed by meiotic or haploid germ cells than in the concentrations of the five of transcripts expressed by SSCs (Fig 1 and S2 Fig). This suggests that SSCs are present in some apparently barren tubules in SCO testes, but produce few differentiated progeny.

### Identification and categorization of human Sertoli cell signature transcripts

In order to define Sertoli cell signature transcripts, we next subjected published data on the transcriptomes of isolated testicular cells to the two-step screen that is described in Methods. The first step used the published transcriptomes of adult rat Sertoli cells, Leydig cells and spermatogenic cells to identify 539 transcripts that were expressed at least 4-fold higher by Sertoli cells than any other cell types in the rat testis [8, 24] (S1 Table). An additional 84 transcripts were added to the tentative Sertoli cell list, even though they were not detected by the arrays, but are known to encode components of the blood-testis barrier or are growth factors for receptors expressed by human SSCs [29, 30]. The second step estimated the expression of these 623 transcripts by human Sertoli cells and compared these estimates to the expression of those transcripts by human Leydig cells and/or by spermatogenic cells [9–12]. We then used three criteria to define a Sertoli cell signature transcript: (a) Sertoli cells were the source of at least 60% of that transcript in the testis, (b) the expression of the transcript by Sertoli cells was at least twice that of Leydig cells, and (c) expression by Sertoli cells was greater than expression by any germ cell. Two hundred forty three transcripts met these criteria. The gene symbols for these transcripts and the calculations that identified them as Sertoli cell signature transcripts are presented in S2 Table.

As discussed in Methods, we predicted that given the important and conserved functions of Sertoli cells, the transcriptomes of mouse Sertoli cells should contain orthologues of most human Sertoli cell signature transcripts. It was therefore of note that 211 of the 243 human Sertoli cells signature transcripts were detected when mature mouse Sertoli cells were subjected to single cell sequencing (S3 Table) [25]. There is precedence for a small difference in the transcriptomes of human and rodent Sertoli cells. In rodent testes Sertoli cells express the sex hormone binding globulin gene [24, 31]; however, in the human testis this gene is not expressed by Sertoli cells but rather by spermatogenic cells [32]. Nonetheless, after we made an overall comparison of the expression of Sertoli cell signature transcripts in testes with complete spermatogenesis with testes with SCO syndrome, we focused our analyses on the human signature transcripts that are also expressed by mature mouse Sertoli cells.

**Fig 2:**
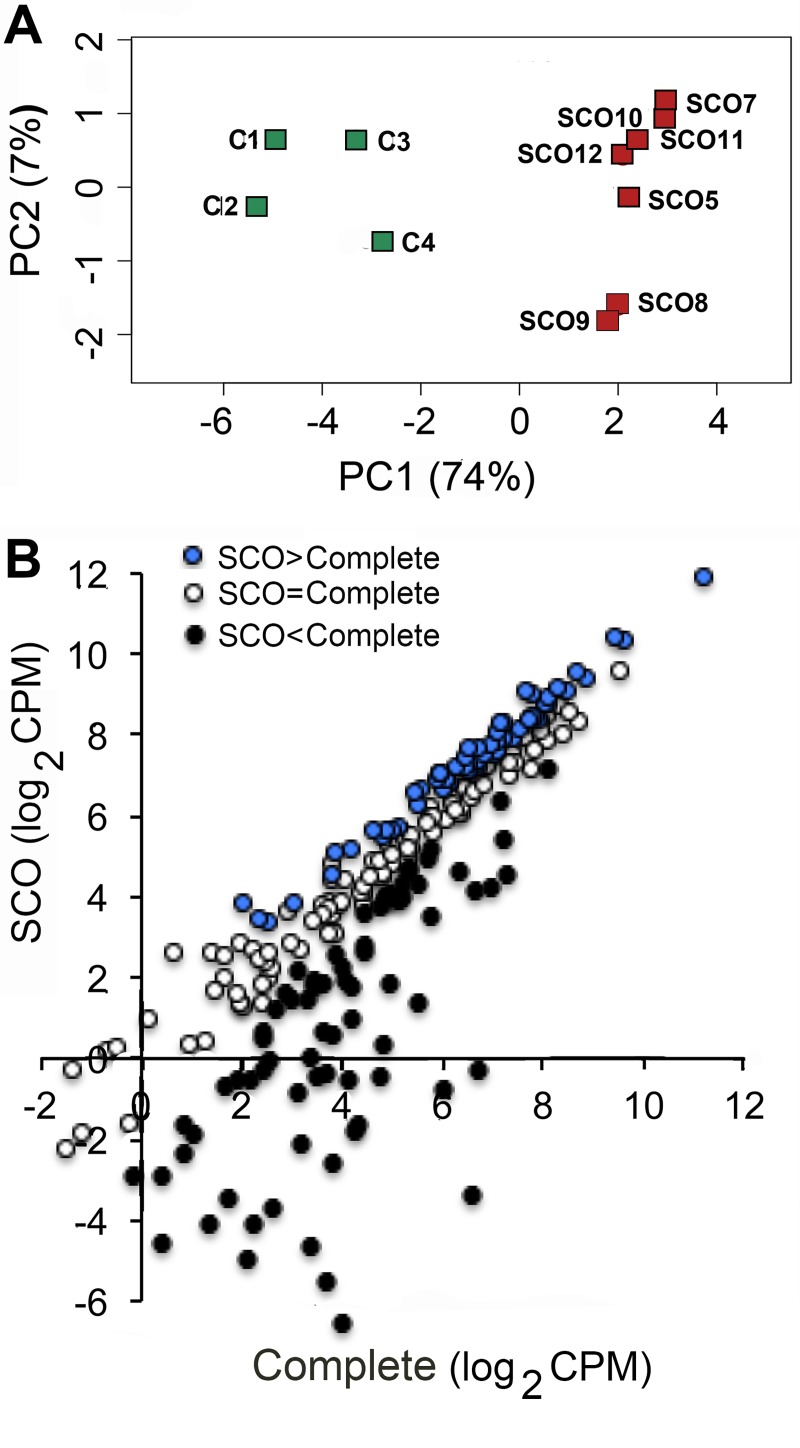
The expression of Sertoli cell signature transcripts in testes with complete spermatogenesis and in testes with SCO syndrome. **(**A) PCA analysis of the expression of Sertoli cell signature transcripts in individual testes. Data were obtained from 4 testes with complete spermatogenesis (C1-4, green symbols) and seven SCO testes (SCO5, SCO7-12, red symbols) (B). A comparison of the mean level of expression of each Sertoli cell signature transcript in testes with complete spermatogenesis and in testes with SCO syndrome. Transcripts that were expressed in SCO testes at significantly higher levels (FDR<0.05), the same level or significantly lower levels are identified by blue, white and black dots, respectively.

Our next step was to identify Sertoli cell signature transcripts whose expressions differed between testes with complete spermatogenesis and testes with SCO syndrome. Expression of these transcripts were normalized to the same median CPM value and compared statistically (S3 Table). Principal component analyses demonstrated that overall, the expression of signature Sertoli cell transcripts differed between the two groups of testes (Fig 2A). Seventy-five and 58 Sertoli cell signature transcripts were significantly more or less abundant, respectively, in SCO testes (Fig 2B). It is noteworthy that four transcripts (WT1, DHH, DKK3 and PRND) that were expressed at higher levels in human SCO testes are essential for fertility of male mice [33–36] (S3 Fig). The remaining 111 signature transcripts were expressed at similar levels in testes with complete spermatogenesis and testes with SCO syndrome. Gene ontology analysis identified some of the less abundant transcripts as associated with cell development or signaling, while more abundant transcripts were associated with actin filaments and non-membrane bound organelles. Thus, this rigorous transcriptional analysis identified subsets of transcripts that could be analyzed further, considering the known characteristics of the SCO testis and its dysfunctional spermatogonial stem cell niche.

### Expression of genes encoding cell surface adhesion proteins and the gap junction protein, Connexin 26

Sertoli cells not only provide the architectural framework for the stem cell niche but also for the entire seminiferous epithelium. In normal testes this epithelium is divided into two compartments, a basal compartment in which spermatogonia and early preleptotene spermatocytes reside and an adluminal compartment that contains more mature spermatogenic cells. The blood-testis barrier separates these two compartments, which are formed by two different types of junctions on apposing Sertoli cell surfaces: adhesive junctions (tight junctions, ectoplasmic specializations (ES)) and gap junctions (communicating junctions), which allow passage of small second messenger molecules, such as cAMP and Ca^+2^, between cells [37]. The same junctions also mediate physical and physiological interactions between Sertoli cells and germ cells, including spermatogonia [38]. It is, therefore, of note that human SCO testes lack a functional blood-testis barrier and that some proteins that mediate adhesion or communication between two adjacent Sertoli cells and between Sertoli cells and spermatogenic cells are either mislocated or undetectable in human SCO testes [13, 14].

**Fig 3:**
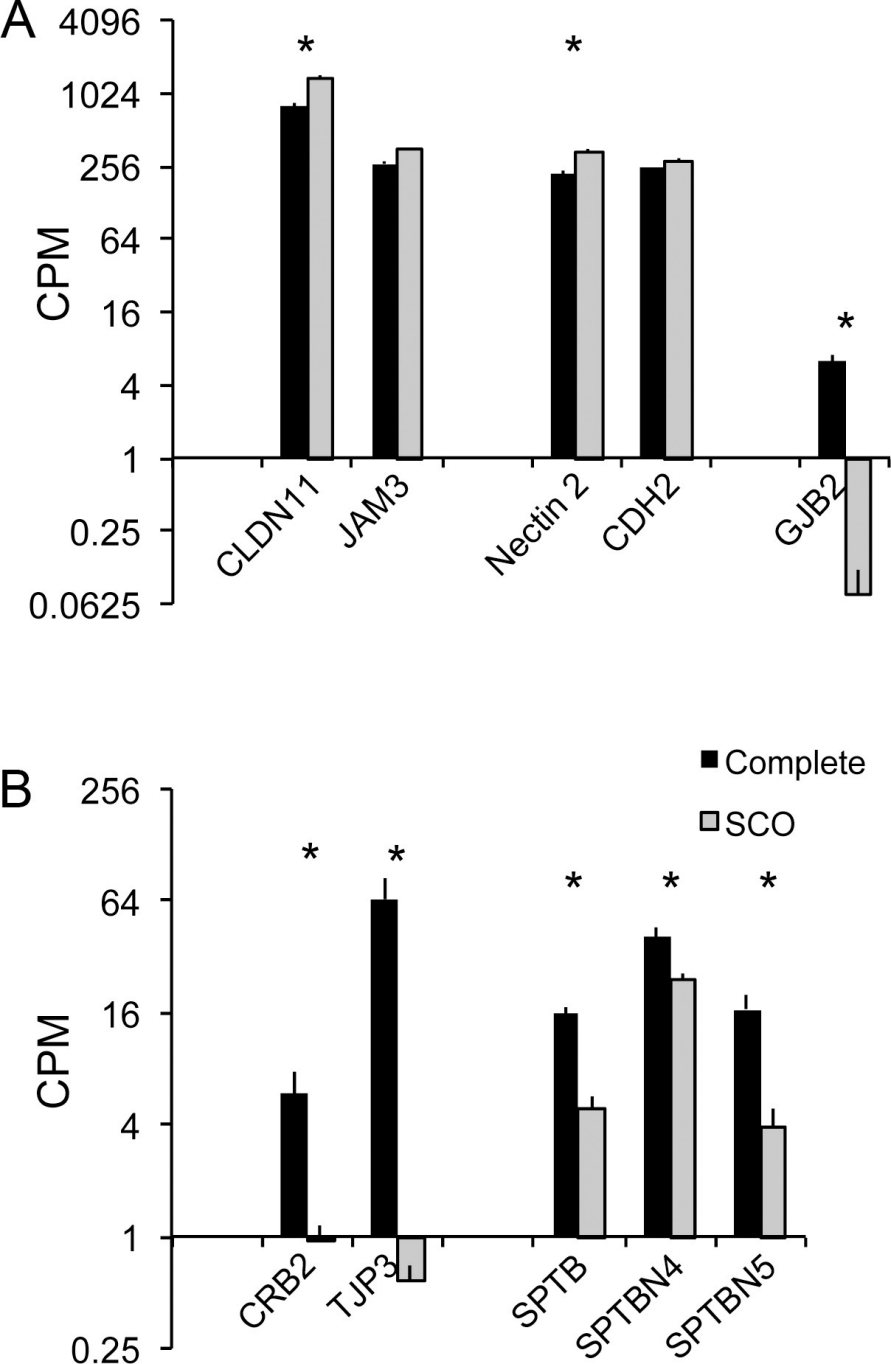
Expression of Sertoli cell signature transcripts encoding proteins that form adhesive or gap junctions or that organize and polarize the Sertoli cell plasma membrane. (A) CLDN11 and JAM3 are tight junction adhesive proteins. Nectin2 and CDH2 are adhesive proteins of ectoplasmic specializations. GJB2 encodes the gap junction protein, Connexin 26. (B) Transcripts that encode proteins that organize or polarize the plasma membrane. CRB2 is a cell polarity component protein that defines the site of separation of the apical and basal-lateral plasma membrane domains. TJP3 is an adaptor protein that links the adhesive proteins of a tight junction to the actin cytoskeleton. SPTB, SPTPN4 and SPTPN5 are scaffolding proteins that concentrate beneath the plasma membrane, seed formation of membrane microdomains, and link those domains to the actin cytoskeleton. All data in this figure are expressed as mean + SEM of CPMs. The presence of an asterisk over a pair of bars indicates a significant difference between testes with complete spermatogenesis and testes with SCO syndrome (FDR≤0.05).

As in other species, human Sertoli cells transcriptionally express multiple integral membrane proteins that form tight junctions and ectoplasmic specializations (ES) [24]. Sertoli cell signature transcripts encode claudins or junction adhesion molecules, adhesive proteins in tight junctions, and cadherins or nectins, the adhesive proteins in ectoplasmic specializations (S2 Table). Fig 3A presents data for the two most abundant transcripts encoding adhesive proteins in tight junctions, Claudin 11 (CLDN 11) and junction adhesion molecule 3 (JAM3), and the most abundant transcripts for the adhesive proteins in ectoplasmic specializations, nectin cell adhesion molecule 2 (NECTIN 2) and cadherin 2 (CDH2). CLDN11 and Nectin 2 were expressed at higher levels in SCO testes; Jam3 and CHD2 were expressed at similar levels in testes with complete spermatogenesis and testes with SCO syndrome. In fact, 8 of the 13 transcripts that encode other adhesive proteins were either expressed at the same or higher levels in SCO testes (S2 Table). Thus, failure of adhesive junction formation is not due to a lack of expression of transcripts that encode the integral membrane adhesive proteins at cell-cell junctions.

We note, however, that not all genes for proteins that mediate cell-cell interactions were expressed at normal or elevated levels in SCO testes. The expression of GJB2, which encodes the gap junction protein connexin 26, was markedly reduced in SCO testes (Fig 3A).

### Reduced transcriptional expression of proteins that polarize and organize the plasma membrane

Although CLDN11 expression is increased in SCO testes, the protein is not concentrated at the presumptive sites of the blood-testis barrier [39, 40]. Rather, most is distributed across the entire Sertoli cell plasma membrane, indicating that these cells lack normal apical-basal polarity and/or membrane microdomains that are required to concentrate adhesive proteins at sites of cell-cell contact [41]. One probable cause for loss of polarity in these cells is inadequate expression of Crumbs (Crb) polarity complex proteins that mark the boundary between the apical and basal-lateral plasma membrane and adaptor proteins (TJPs) that stabilize the tight junctions assembled at that site [41]. Sertoli cell signature transcripts encode CRB2 and TJP3, and their expression in SCO testes were reduced to 16% and 1%, respectively, of their expression in testes with complete spermatogenesis (Fig 3B). We recognize that other polarity and adaptor proteins, CRB3, TJP1 and TJP2, have been implicated in formation of the blood-testis barrier by rodent Sertoli cells [42, 43]. While we did not define these 3 proteins as Sertoli cell signature transcripts, it should be noted that when the abundance of their transcripts in total testis transcriptomes was normalized to beta actin mRNA, expressions of all three were significantly reduced in SCO testes (S4 Fig). Thus, markedly reduced expression of CRB2 and TJP3 in SCO testes is not compensated by increased expression of other polarity and adaptor proteins.

The localization of adhesive and gap junctions within sites in the plasma membrane that abut other cells requires a higher level of membrane organization than is achieved by separation of the apical from the basal-lateral plasma membrane. Proteins in the spectrin family of actin-binding proteins play a key role in the formation of plasma membrane microdomains and the concentration of junctional molecules in those domains [44, 45]. Three Sertoli cell signature transcripts encode the spectrins, SPTB, SPTBN4, and SPTBN5, which were reduced in expression from 23% to 60% in SCO testes (Fig 3B). These decreases and those for transcripts encoding CRB2 and TJP3 lead us to conclude that in SCO testes Sertoli cells can form neither a barrier that separates the apical and basal-lateral plasma membrane nor the membrane microdomains that normally concentrate cell adhesion complexes at sites of cell-cell contact.

### Reduced expression of genes that act in pathways, which regulate vesicular trafficking

**Fig 4:**
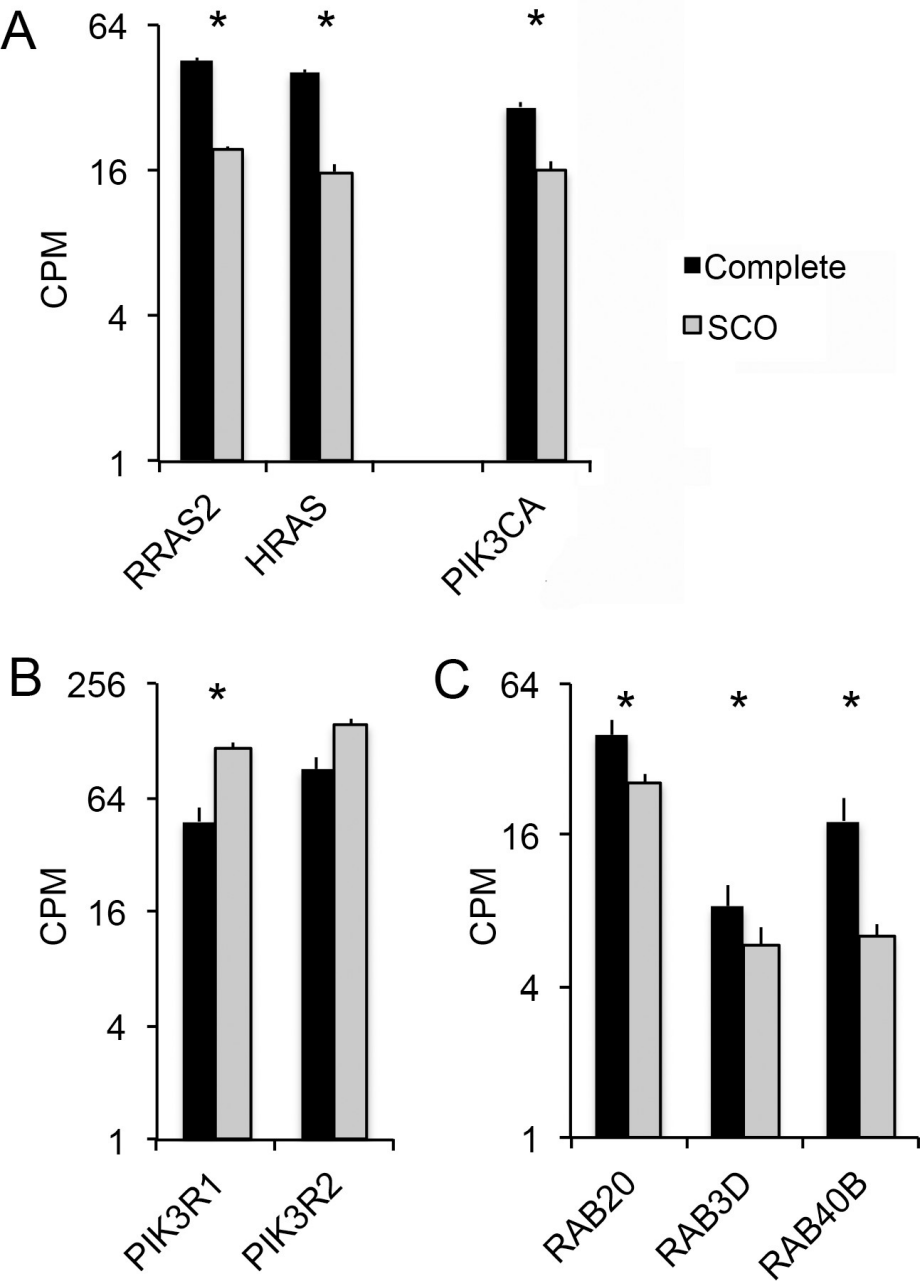
Expression of Sertoli cell signature transcripts encoding regulators of vesicular trafficking. (A) Expression of RRAS2 and HRAS and the catalytic subunit of phosphoinositide 3-kinase (PI3K), PIK3CA. Binding of RRAS2 and HRAS to PI3KCA stimulates its catalytic activity. (B) Expression of regulatory subunits of PI3K. (C) Small GTPase family members that regulate vesicular trafficking. RAB20 regulates endocytic trafficking; RAB40B and RAB3D regulate exocytic trafficking. Activities of these small GTPases are stimulated by PI3K. Data are expressed as mean + SEM of CPMs for each transcript. The presence of an asterisk over a pair of bars indicates a significant difference between testes with complete spermatogenesis and testes with SCO syndrome (FDR<0.05).

An essential requirement for male fertility is the dynamic assembly and disassembly of junctions between adjacent Sertoli cells and between these somatic cells and spermatogenic cells [46, 47]. This dynamism requires vesicular trafficking of membrane proteins in and out of the plasma membrane [48]. This trafficking is regulated by a pathway that begins with the binding of RAS GTPases to catalytic subunits of phosphoinositide 3-kinase (PI3K) [49]. The resulting activation of PI3K enzyme activity stimulates RAB GTPases, the immediate regulators of exocytic and endocytic vesicular trafficking [15, 50]. Sertoli cells within SCO testes expressed markedly reduced levels transcripts that encode key enzymes in this pathway. Expressions of RRAS2 and HRAS mRNAs were reduced to 40% of what we measured in testes with complete spermatogenesis; expression of the catalytic subunit of PI3K, PI3CA, was reduced by 50% (Fig 4A). In contrast, expression of two PI3K regulatory subunits, PI3KR1 and PI3KR2, were increased or similar to expression in testes with complete spermatogenesis (Fig 4B). Multiple RABS encoded by Sertoli cell signature transcripts were also significantly reduced in SCO testes (Fig 4C and S3 Table). For example, SCO testes expression of RAB20, a regulator of endocytic vesicular trafficking, was reduced by 65% [51], while expression of the regulators of exocytic vesicles, RAB3D and RAB40B, were reduced by 60% and 70%, respectively [52, 53]. Thus, Sertoli cells within SCO testes exhibit significant deficits in expression of multiple genes that act at different points in the pathway that regulates the vesicular trafficking of junctional proteins to and from the plasma membrane.

In summary, we conclude that there are at least three reasons that Sertoli cells in SCO testes cells do not form normal cell-cell junctions with each other and with spermatogenic cells, including SSCs: First, these somatic cells are unable to form a boundary that is required for the separation of the apical from the basal-lateral plasma membrane. Thus, the plasma membrane is not properly polarized. Second, these cells do not form the proper membrane microdomains into which adhesive proteins and communicating junctions are normally concentrated. Third, there is inefficient vesicular trafficking of proteins that form adhesive and communicating junctions, thereby suppressing the formation and turnover of junctions that tether Sertoli cells to each other or to any neighboring spermatogenic cells.

### Deficient expression of three growth factors that regulate replication and differentiation of SSCs

There is considerable evidence from studies of mice that multiple growth factors regulate the size and function of the spermatogonial stem cell pool [5]. Human SSCs undoubtedly share this requirement for they express receptors for all of the growth factors that are required by mouse SSCs [30]. Therefore, it follows that inadequate stimulation by growth factors may be a significant contributor to SCO syndrome.

**Fig 5:**
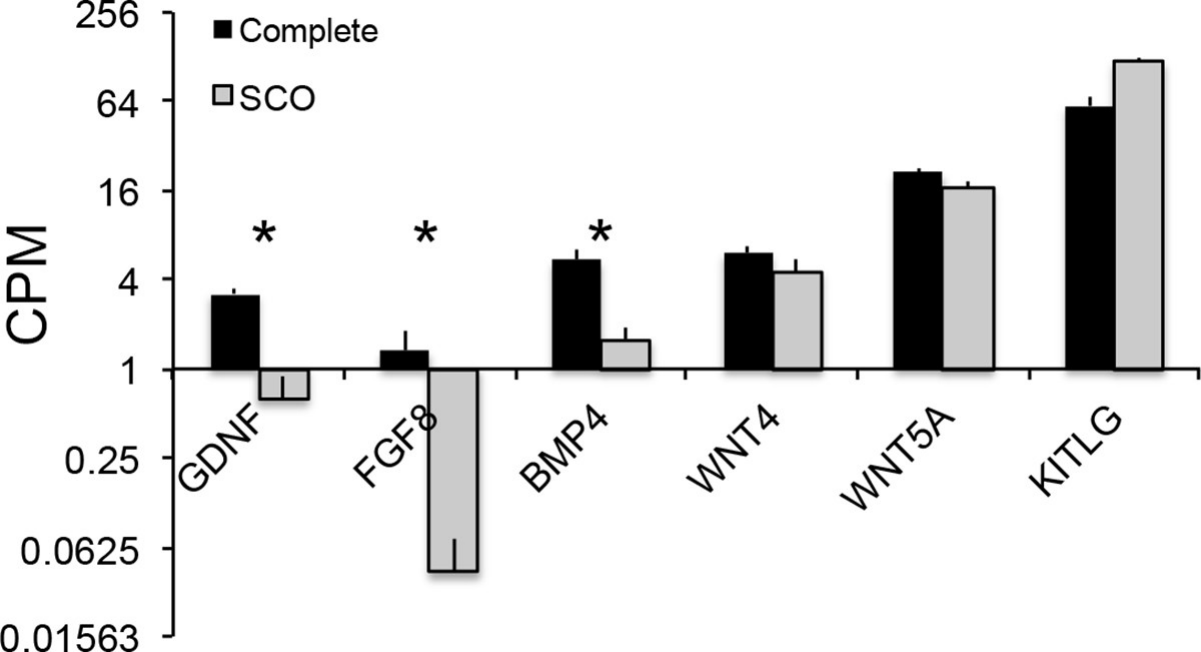
Expression of Sertoli cell signature transcripts that encode growth factors that are known regulators of SSCs and/or progenitor spermatogonia. Data are expressed as mean + SEM of CPMs. An asterisk over a pair of bars indicates a significant difference (FDR≤0.05).

Our analyses identified 19 Sertoli cell signature transcripts that encode growth factors or cytokines that are ligands for those receptors (S2 Table). Five factors, GDNF, FGF8, BMP4, Wnt4 and Wnt5A, have defined effects on mouse SSCs and/or progenitor spermatogonia [3, 6, 54–57]. Expressions of 3 of these growth factors were significantly reduced in SCO testes (Fig 5). Consistent with our earlier report [19], expression of GDNF in SCO testes was only 20% of expression in testes with complete spermatogenesis. FGF8 mRNA expression was also markedly reduced in SCO testes. Our analyses indicated that there was no compensation for this marked decrease in FGF8 mRNA by increased transcriptional expression of another member of the fibroblast growth factor family, FGF2, which binds the same receptor as FGF8 [29]. While FGF2 is not encoded by a Sertoli cell signature transcript, it is worth noting that when the abundance of FGF2 mRNA in the total testis transcriptome was normalized to beta actin mRNA, expression of FGF2 mRNA was significantly reduced in SCO testes (S5 Fig).

BMP4, WNT4 and WNT5A have distinct effects on mouse SSCs; BMP4 and WNT4 promote SSC differentiation, while WNT5A promotes stem cell maintenance [55, 57–59].

Since human SSCs express receptors for these three growth factors [30], it is probable that they have similar effects on human SSCs. Our analyses demonstrated that in SCO testes BMP4 mRNA expression was reduced to 30% of expression in testes with complete spermatogenesis. In contrast, the two WNT family members were expressed at similar levels (Fig 5). So, too, was an important regulator of the final steps in spermatogonial differentiation, kit ligand (KITLG) [60] (Fig 5).

CSF1 and CXCL12 are two additional growth factors that are known to regulate mouse SSCs [61, 62]. While they were not identified as Sertoli cell signature transcripts, it should be noted that examination of the total testis transcriptomes revealed that beta actin-normalized expression of CSF1 and CXCL12 mRNA was similar in normal and SCO testes (S5 Fig).

**Fig 6:**
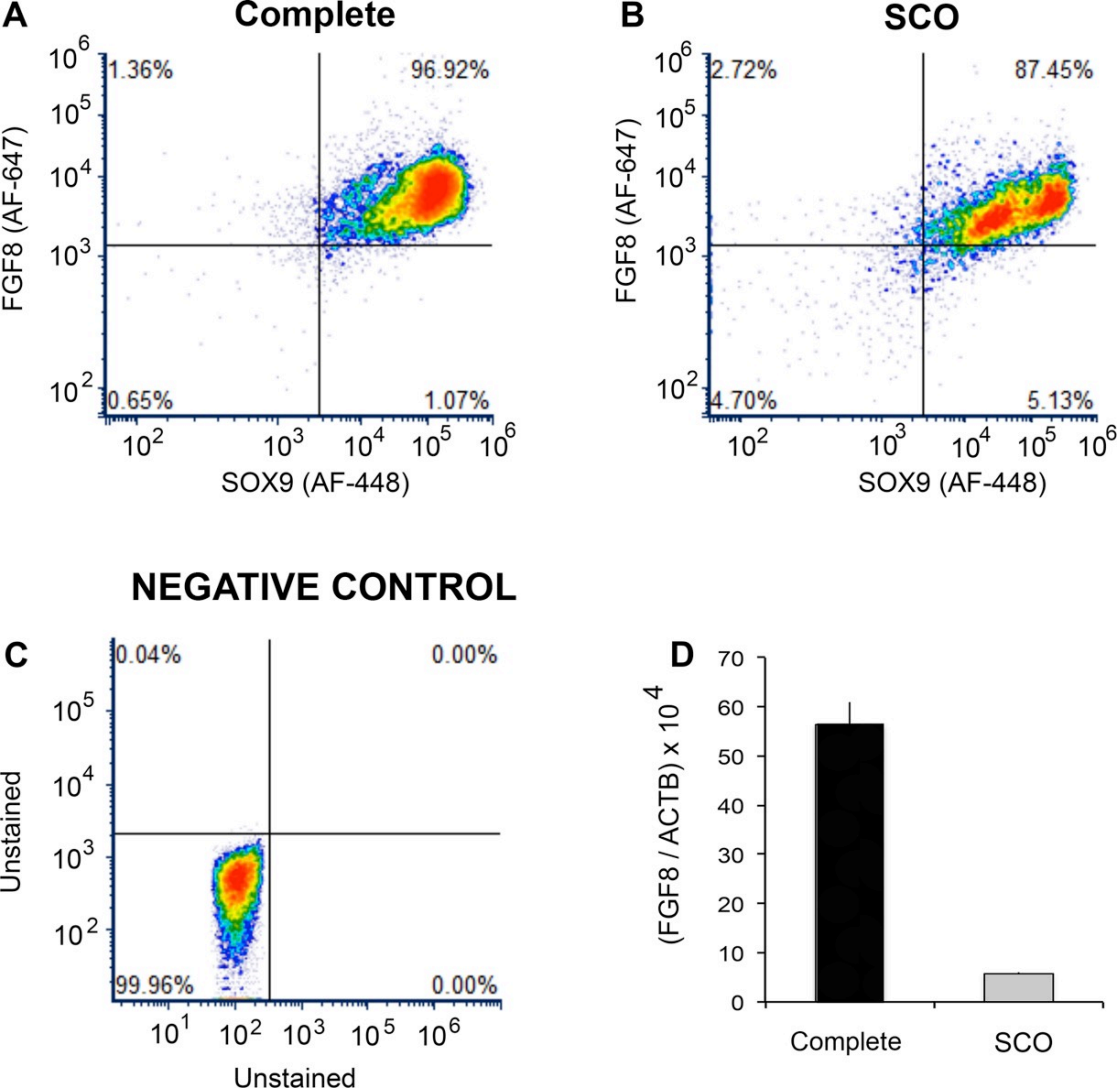
Confirmation that FGF8 is a product of human Sertoli cells and verification that expressions of both FGF8 protein and mRNA are reduced in SCO testes. Testes with complete spermatogenesis (A) and with SCO syndrome (B) were dispersed into single cells and then incubated with antibodies for the Sertoli cell marker SOX9 and for FGF8. Negative control cells (C) were not incubated with antibodies. Cells were then analyzed FACs. These data are representative of three independent analyses. (D) Quantification by real-time PCR of levels of FGF8 mRNA in biopsies of human testes with complete spermatogenesis and in testes with SCO syndrome. Data (mean + SEM; n = 3) were normalized for expression of beta actin in the same sample (p<0.001).

While our screening process identified FGF8 as a Sertoli cell signature transcript, studies of mice concluded that spermatogenic cells and not Sertoli cells are this growth factor’s source [56]. Given the almost complete lack of expression of FGF8 in SCO testes and its well-characterized effect on mouse SSCs, we performed additional experiments to prove that FGF8 was a product of human Sertoli cells, and we examined whether FGF8 protein content of SCO Sertoli cells was significantly diminished. FACs analysis demonstrated that all cells that express FGF8 also express SOX9, a specific marker of Sertoli cells [63] (Fig 6A). Furthermore, while in human testes with complete spermatogenesis FGF8 expression per Sertoli cell exhibited a normal distribution, FGF8 protein expression in SCO testes was bimodal with approximately 50% of Sertoli cells expressing abnormally low levels (Fig 6B). However, those low levels of FGF8 were substantially greater than background in this assay (Fig 6C). Finally, quantitative PCR analysis confirmed that FGF8 mRNA expression was markedly reduced in SCO testes (Fig 6D). Thus, the SSCs that are present in some human SCO testes are deprived of two growth factors (GDNF and FGF8) that promote”stemness” and one factor (BMP4) that stimulates differentiation. This suggests that the regulation of the balance between self-renewing stem cell replication and stem cell differentiation is askew.

## Discussion

**Fig 7:**
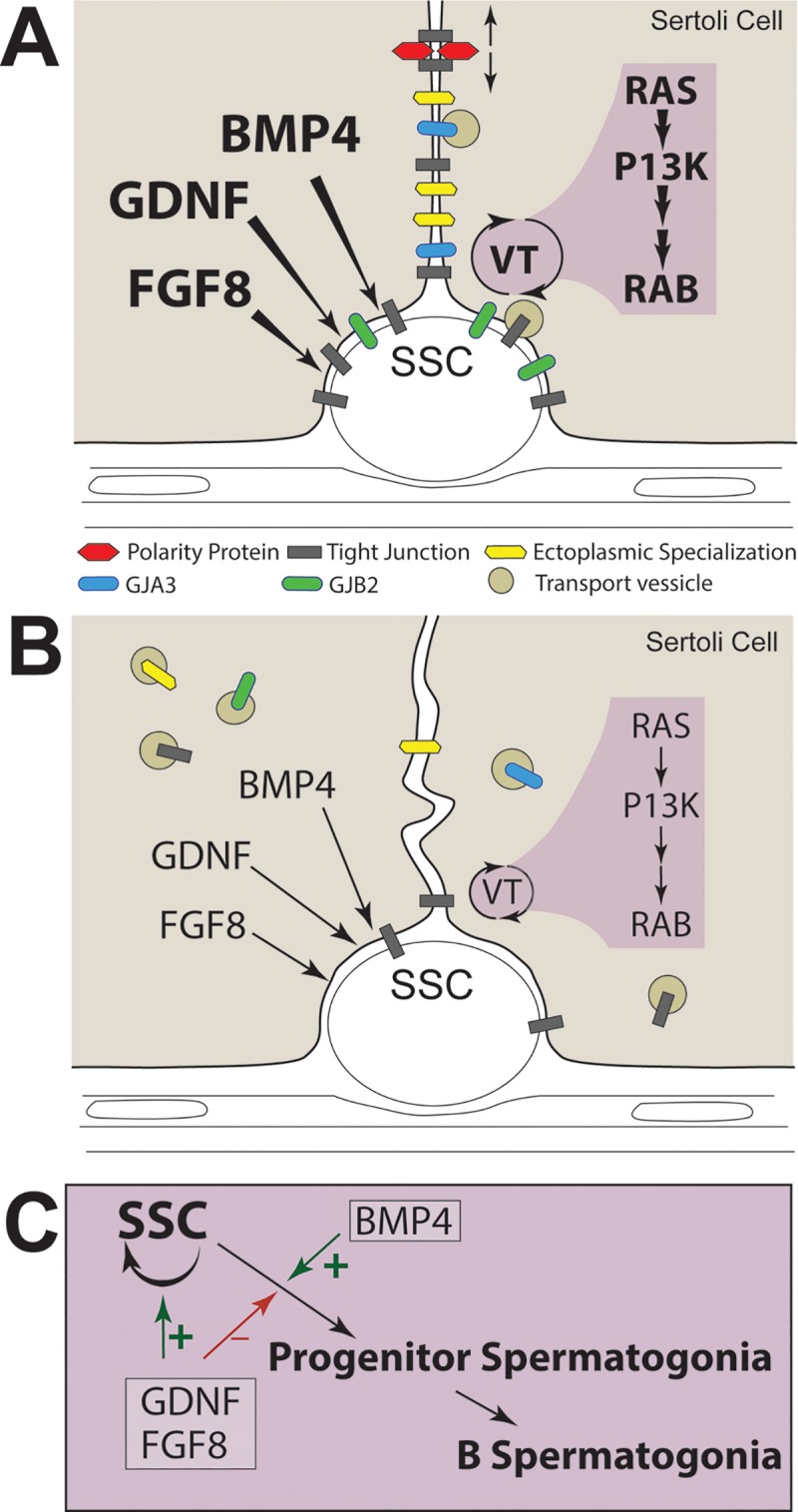
Summary: Deficits in Sertoli cell gene expression that are associated with specific aspects of human SCO syndrome. (A) Human testis with complete spermatogenesis. Polarity complex proteins (red) and tight junctions (gray) polarize the plasma membrane thereby generating apical and basal-lateral domains (identified by up and down arrows). Additional tight junctions, ectoplasmic specialization (yellow) and gap junctions (blue and green) are concentrated at sites of cell-cell contact. Vesicular trafficking (VT; tan circles) of adhesive and gap junction proteins facilitates the assembly and disassembly of junctions, controlled by a pathway that includes Ras GTPases, PI3K and RAB small GTPases (purple shading). Sertoli cells express normal amounts of GDNF, FGF8 and BMP4, growth factors that regulate replication and differentiation of SSCs. (B) The infertile SCO testis. Inadequate expression of polarity and adaptor proteins renders Sertoli cells incapable of properly polarizing their plasma membranes. There is reduced, inefficient vesicular trafficking of proteins that form tight junctions, ectoplasmic specializations and gap junctions, stranding these proteins in vesicles within the cytoplasm. Finally, there is reduced expression of GDNF, FGF8 and BMP4. (C) The effects of GDNF, FGF8 and BMP4 on SSCs and progenitor spermatogonia. GDNF and FGF8 promote self-renewing replication of SSCs, while BMP4 stimulates differentiation of SSCs into progenitor spermatogonia and progenitors into fully differentiated B spermatogonia.

There is considerable evidence that a fertile testis is characterized by precise and complex interactions between testicular somatic cells and spermatogenic cells [24, 64]. One result of these interactions is the formation of a functional niche for spermatogonial stem cells by Sertoli and other somatic cells. It follows that aberrant gene expression by Sertoli cells might decrease niche function and contribute to the dearth and dysfunction of SSCs in infertile men with SCO syndrome. Furthermore, niche dysfunction might also explain why the few SSCs in tubule segments with active spermatogenesis do not replicate and fill empty niches within SCO testes. This first thorough comparison of the transcriptome of testes with complete spermatogenesis with the transcriptome of testes with SCO syndrome was motivated by the possibility that defining the transcriptional basis of niche dysfunction would be an important first step to development of new therapies for men with SCO syndrome whose testes contain small segments of tubules with active spermatogenesis. We have focused our analyses on 244 transcripts, which our two-step screen identified as human Sertoli cell signature transcripts. Two hundred eleven of these are also expressed by mature mouse Sertoli cells. Such signature transcripts are a foundation of these cells’ critical but varied functions, and one such function is the creation of the niche for SSCs. Our analyses demonstrated that 69% of these transcripts were expressed at normal or elevated levels in SCO testes. However, 31% were expressed at significantly lower levels and their decreased expression provides probable explanations for two important characteristics of SCO testes. As summarized in Fig 7, we conclude that the failure of Sertoli cells in SCO testes to form normal cell-cell junctions results from their inability to assemble and stabilize those junctions at normal sites of cell-cell contact. We also conclude that Sertoli cells within SCO testes express abnormally low levels of GDNF, FGF8 and BMP4, thereby depriving SSCs of growth factors that have been well characterized as regulators of mouse SSCs.

### Failure of Sertoli cells to form normal cell-cell junctions

The failure of Sertoli cells in SCO testes to form normal cell-cell junctions is not due to inadequate expression of transcripts that encode the adhesive proteins within those junctions. Our studies corroborated earlier reports that these somatic cells express elevated levels of the tight junction protein claudin 11 in SCO testes [65]. A new finding from our analyses, however, is that these Sertoli cells express normal or elevated levels of transcripts encoding many other major integral membrane adhesive proteins. Moreover, our analyses identified deficits in the expression of genes that polarize the plasma membrane or concentrate adhesive junctions in the microdomains of plasma membranes that abut other cells. Furthermore, our data identified markedly reduced expression of genes in a pathway that regulates vesicular trafficking of junctional proteins in and out of the plasma membrane.

In SCO testes much of the tight junction adhesive molecule, claudin 11, is dispersed across the entire Sertoli cell plasma membrane rather than at the presumptive sites of the blood-testis barrier [40]. This dispersal indicates that the cells have failed to segregate their plasma membranes into apical and basal-lateral domains. Our data identified deficits in expression of two genes that are the likely causes of this failure, the Crumbs (Crb) polarity complex component CRB2 and the adaptor protein, TJP3. Both normally play important roles in membrane polarization. The insertion of CRB2 into the plasma membrane marks the site at which a barrier between the apical and basal-lateral plasma membrane is constructed [41]. This insertion also recruits tight junction adhesive proteins, such as CLDN11 and JAM3, which are then anchored to the actin cytoskeleton by adaptor proteins, such as TJP3. In the absence of Crb proteins, tight junction formation remains incomplete and lacks barrier function [41]. Without adaptor proteins tight junction adhesive proteins are not anchored at the presumptive site of the barrier [66]. The expression of CRB2 and TJP3 was significantly reduced in SCO testes, and the lack of compensatory increases in expression of other CRBs or adaptor proteins makes it highly probable that Sertoli cells nether efficiently mark the site in the plasma membrane for barrier formation nor anchor tight junctions at that site. Unmoored, tight junction adhesive proteins would, thus, be dispersed across the entire Sertoli cell surface. Studies of human SCO testes have reported such dispersion [40]. This lack of membrane polarity would also place fewer constraints on the distribution of all other junctional molecules within the plasma membrane.

Our analyses indicated that the effects of diminished plasma membrane polarity on the formation of cell-cell junctions are likely further exacerbated by a significantly reduced expression of three members of the spectrin-family of actin-binding proteins, SPTB, SBTBN4 and SPTBN5. Spectrins play a key role assembling lipid rafts within a plasma membrane and in concentrating multiprotein adhesion and signaling complexes within those rafts [44, 45, 67, 68]. Thus, reduced expression of these three spectrins synergizes with inadequate expression of CRB2 and TJP3 to disrupt the ability of Sertoli cells to form adhesive junctions with each other and with spermatogenic cells, including SSCs.

### Reduced expression of genes in pathways that regulate vesicular trafficking

In a normal testis, interactions between adjacent Sertoli cells and between Sertoli cells and germ cells, including spermatogonia, are dynamic. Movement of preleptotene spermatocytes into the adluminal compartment of the seminiferous tubule requires simultaneous elimination of Sertoli-Sertoli junctions above the germ cells and assembly of new junctions below [47, 69]. Concurrently, junctions that hold elongate spermatids to Sertoli cells are disassembled in preparation for spermiation [70]. Vesicular trafficking is responsible for assembling and disassembling cell-cell junctions, and phosphoinositide 3-kinase (PI3K) and the pathway in which it acts are key traffic regulators [50]. As binding of HRAS and RRAS-2 to PI3K’s catalytic subunits stimulates PI3K activity [49], the markedly reduced expression of HRAS and RRAS-2 and of PI3CA must greatly diminish Sertoli cell PI3K enzyme activity in Sertoli cells within SCO testes. We suggest that coupling this reduction with significantly lower expression of some RAB GTPases decreases the efficiency of vesicular trafficking to and from the plasma membrane, leaving some vesicles and the junctional proteins they contain stalled in the cytoplasm. This suggestion is supported by the report that in SCO testes some claudin 11 is located in the cytoplasm of Sertoli cells [71]. It follows that inefficient trafficking further reduces the ability of these Sertoli cells to assemble adhesive and communicating junctions at sites of cell-cell contact.

### Connexin 26

An unexpected finding from our analyses was that Sertoli cells in SCO testes expressed markedly reduced levels of connexin 26 (GJB2). In rodents connexin 26 is concentrated at sites where Sertoli cells and spermatogonia abut [37]. It is, therefore, of interest that rodent Sertoli cells communicate efficiently via gap junctions with spermatogonia, and spermatocytes [72, 73]. While GJB2 is also expressed by human spermatogonia (S1 Table), the reduction in GJB2 expression to 2% of what is observed in testes with complete spermatogenesis suggests that there is reduced gap junctional communication between Sertoli cells and the few SSCs that exist in an SCO testis.

### The importance of cell adhesion to SSCs

Since Sertoli cells form adhesive junctions with spermatogonia [40], one must consider the possible consequence of loss of cell adhesion to SSCs. First, this loss will reduce signaling by focal adhesion kinases within stem cells [74]. Second, decreased adhesive strength deprives SSCs of the positional information they need to divide asymmetrically. (Studies of pig SSCs document such divisions [16]). Asymmetric division is a common strategy that adult stem cells use to balance generation of new stem cells with the production of differentiating progeny [16, 75, 76]. Thus, the loss of positional information in SCO testes reduces the probability of asymmetric division by SSCs, thereby threatening the maintenance of the stem cell pool.

### Deficient expression of growth factors that regulate SSCs

Replication and differentiation of SSCs are regulated by multiple growth factors, and our analyses demonstrated that GDNF, FGF8 and BMP4 were expressed at abnormally low levels in SCO testes. All three are well-characterized regulators of mouse SSCs and progenitor spermatogonia [6, 56, 58, 59]. Sertoli cells are the only major source of GDNF in the human testis, and between the current and previous studies [19], we have evaluated GDNF expression in 21 separate human SCO testes. Here and in our previous study, the average GDNF mRNA level in SCO testes was reduced to approximately 20% of normal, and all SCO testes expressed lower levels of GDNF than testis with complete spermatogenesis. Studies in mice suggest that as little as a 50% reduction in GDNF expression is sufficient to cause gradual development of SCO syndrome [77]. Thus, it is becoming increasingly clear that inadequate GDNF expression by Sertoli cells is a fundamental characteristic, as well as one probable cause, of human SCO syndrome.

Our studies led to important new conclusions: human Sertoli cells are the predominant testicular source of FGF8, and expression of this growth factor is less than 10% of normal in SCO testes. Additionally, our data indicate that there is no compensatory increase by SCO testes in FGF2 expression, which binds the same receptors as FGF8 [29]. The probable consequence of this 95% decrease in FGF8 expression is evident in studies of mice. FGF8 suppresses the early steps in differentiation of SSCs and early progenitor spermatogonia, thereby promoting the maintenance of a normal stem cell pool [56]. Thus, studies of mouse SSCs predict that the greatly diminished expression of both GDNF and FGF8 in SCO testes deprives these stem cells of stimuli that both promotes self-renewing replication and suppresses differentiation of these stem cells. Furthermore, loss of adequate GDNF stimulation deprives the few remaining SSCs of chemotactic stimuli that would normally drive these stem cells to migrate to empty niches [18, 78]. BMP4 is the third growth factor that we identified as expressed at substantially lower levels in SCO testes. Studies of mice indicate that this growth factor stimulates stem cells to form progenitor spermatogonia and progenitors to express Kit, the receptor for Kit ligand, a growth factor that drives the final steps in differentiation of human progenitor spermatogonia into Type B spermatogonia [3, 58]. Thus, while Kit ligand expression was at normal levels in SCO testes, its effect on progenitors is probably dampened by the lack of BMP4-driven Kit expression by these cells. Thus, the reduced expression of BMP4 provides one explanation for our observation that there is a substantially greater decrease in expression of markers for spermatocytes and spermatids than for SSCs in SCO testes.

### Next steps to a full understanding of Sertoli cell-only syndrome

This study described herein is but a first step towards a full understanding of the molecular basis of Sertoli cell-only syndrome. It is critical that we attain this understanding. First, Sertoli cell-only syndrome is one of the severest forms of male infertility, which is a major medical and public health problem [1]. Second, small segments of tubules in SCO testes produce sperm, and thus must contain SSCs [2]. Third, there is a pressing need to develop new therapies for human male infertility. Fourth, identifying aberrations in gene expression by SCO Sertoli cells might lead to new approaches to correct or compensate for niche dysfunction, thereby increasing the chances of SCO infertile men becoming biological fathers. The data presented described herein are a necessary first step to defining those aberrations. However, as with all first steps, this one has its limitations. Because the transcriptome of normal adult human Sertoli cells has not been characterized directly, we relied on a rigorous two-step bioinformatic analytical approach to define human Sertoli cell signature transcripts. As we previously noted, all mammalian Sertoli cells perform the same essential functions and it is highly probable that Sertoli cells of different species express orthologous genes in order to perform these functions. Nonetheless, a next important step will be to thoroughly describe the entire transcriptome of highly purified human Sertoli cells that have been rapidly isolated from fertile testes. A prerequisite for this step is the development of the isolation protocol. Rapidity is necessary to minimize changes in these cells’ transcriptomes. A second limitation of the current study is that the small mass and immediate freezing of the biopsies (15–40 mg) precluded the isolation of Sertoli cells prior to the evaluation of their transcriptomes. We anticipate that this second limitation will be circumvented by single cell transcriptome analysis of cells in freshly obtained biopsies. However, clear and compelling scientific rationale is required before such a study can be proposed and conducted. Our analyses described herein build such rationale. Finally, it is likely that a comparison of the transcriptomes of dialated, germ cell-filled tubules and atretic tubules from the same SCO testes would provide significant insights into the transcriptional foundation of SCO syndrome. However, as dialated tubules are potential sources of sperm, and as the clinical objective of the micro-Tese procedure is to help infertile men conceive a child, it is necessary that all dialated tubules be used for sperm retrieval.

We also acknowledge that signals from spermatogenic cells regulate a subset of the genes expressed by Sertoli cells. Thus, some of the aberrations in Sertoli cell gene expression noted herein could be secondary to loss of such regulatory signals [7]. However, we doubt that lack of germ cells is the primary cause of the aberrant gene expression by Sertoli cells in SCO testes. Consider the following: While GDNF mRNA expression is markedly reduced in SCO testes, its expression is markedly increased in germ cell-deficient mouse testes [79]. Additionally, human Sertoli cells in SCO testes do not form a functional blood-testis barrier [13, 14], and our data indicate that they do not express adequate levels of genes required for polarization of their plasma membrane or efficient transportation of cell adhesion proteins to the plasma membrane. In contrast, Sertoli cells in germ cell deficient rat testes form a functional blood-testis barrier [80]. It follows that those rat Sertoli cells must express adequate levels of the genes that are essential for polarization of the Sertoli cell plasma membrane and assembly of junctions at sites of normal cell-contact. Finally, others and we have proven that both stimulatory and inhibitory signals from spermatogenic cells regulate gene expression by Sertoli cells. This complex regulation is clearly evident in the stage-specific changes in rat Sertoli cell expression of the cathepsin L (CTSL) gene [7]. Stimulatory and inhibitor signals from germ cells act via enhancers and repressors in the CTSL gene promoter to regulate stage-specific transcription. Loss of germ cells does not substantially change total testis content of cathepsin L mRNA; rather all Sertoli cells express similar levels of the transcript. Other genes show a similar response to loss of germ cells [7]. Thus, we propose that the aberrant expression of some and possibly many genes by Sertoli cells in SCO testis is a characteristic of the syndrome and is not secondary to the loss of spermatogenic cells.

### Conclusion

The goal of our analyses was to identify changes in gene expression by human Sertoli cells that might explain two characteristics of the dysfunctional stem cell niche in SCO testes and thereby potentially identify some of the mechanistic underpinnings of this infertile human phenotype (Fig 7). The inability of these Sertoli cells to form normal cell-cell junctions can be explained by their inadequate expression of genes required to properly polarize and organize the plasma membrane and by inefficient cycling of proteins that form adhesive and communicating junctions in and out of the plasma membrane. Sertoli cells in SCO testes also express abnormally low levels of three growth factors GDNF, FGF8 and BMP4, which are important regulators of the replication and differentiation of mouse SSCs and progenitor spermatogonia. We propose that throughout most of an SCO testis, GDNF and FGF8 levels are inadequate to stimulate both the replication of SSCs and their migration into germ cell-deficient areas of seminiferous tubules. Low levels of BMP4 may deprive stimulus that normally drives differentiation of SSCs and progenitor spermatogonia. Thus, our data indicate that SCO syndrome is a complex condition associated with multiple deficits in Sertoli cell gene expression. We acknowledge that further studies are needed to gain a more clear and precise understanding of the contribution of Sertoli cells to SCO syndrome. We hope that such an understanding might foster the development of new therapies to stimulate the expansion of the few spermatogonial stem cells that are present some SCO testes, thereby increasing the possibility that an infertile man can become a father.

## Supporting information

S1 Text Supplementary methods. (DOCX)

S1 Fig The histologies of human testes with normal spermatogenesis, with mild hypospermatogenesis and moderate hypospermatogenesis. The testes with these three histologies are all referred to in the manuscript as exhibiting complete spermatogenesis. Photomicrographs were captured using 10X and 40x microscope lenses.
(TIF)

S2 Fig Abundance of transcripts characteristic of SSCs (GFRA1), spermatocytes (SYCP2, SYCP3) and spermatids (PRM1, PRM2) in each of the 4 testes with complete spermatogenesis (C1-C4) and in each of the 7 testes with SCO Syndrome (SCO5, SCO5-12). Data are from total testis transcriptomes and are expressed as CPM for each of the above transcripts divided by CPM for beta actin.
(TIF)

S3 Fig Increased abundance in SCO testes of four Sertoli cell signature transcripts essential for spermatogenesis. Data (mean+ SEM) are expressed CPM. An asterisk over a pair of bars indicates a significant difference between testes with complete spermatogenesis and SCO testes (FDR≤ 0.05).
(TIF)

S4 Fig Abundance of transcripts encoding the cell polarity protein, CRB2 and the adapter proteins TJP1 and TJP2 in the total testis transcriptomes of normal and SCO testes. Data (mean + CPM) are expressed as CPM in the total testis transcriptome divided by CPM of ACTB in the same sample. Asterisks over a pair of bars indicate that normalized expression of a transcript differs between testes with complete spermatogenesis and SCO testes (p≤0.005).
(TIF)

S5 Fig Abundance in the total testis transcriptomes of normal and SCO testes of FGF2, CSF1 and CXCL12, three growth factors that have been demonstrated in studies of mice to regulate SSCs or progenitor spermatogonia. Data (mean + SEM) are expressed as CPM in the total testis transcriptome divided by CPM of ACTB in the same sample. An asterisk over a pair of bars indicates a significant difference between testes with complete spermatogenesis and SCO testes (p≤0.005).
(TIF)

S1 Table Identification of transcripts expressed at least 4-fold higher by rat Sertoli cells than by rat Leydig cells, pachytene spermatocytes, round spermatids and spermatogonia. (XLSX)

S2 Table Definition of human Sertoli cell signature transcripts. (XLSX)

S3 Table Expression of Sertoli cell signature transcripts in testis with complete spermatogenesis and in testes with Sertoli cell-only syndrome. (XLS)
