# Correction: Aberrant gene expression by Sertoli cells in infertile men with Sertoli cell-only syndrome

**DOI:** 10.1371/journal.pone.0221648

**Published:** 2019-08-20

**Authors:** The PLOS ONE Staff

There are errors in the author affiliations. The publisher apologizes for the errors. The correct affiliations are as follows:

Darius A. Paduch^1,2^, Stephanie Hilz^3,4^, Andrew Grimson^3^, Peter N. Schlegel^1^, Anne E. Jedlicka^5^, William W. Wright^6^

**1** Department of Urology, Weill Cornell Medical College, New York, NY, United States of America, **2** Consulting Research Services, Inc, North Bergen, N.J., United States of America, **3** Department of Molecular Biology and Genetics, Cornell University, Ithaca, NY, United States of America, **4** Department of Neurological Surgery, University of California, San Francisco, California, United States of America, **5** Genomic Analysis and Sequencing Core Facility, Johns Hopkins Bloomberg School of Public Health, Baltimore, Maryland, United States of America, **6** Department of Biochemistry and Molecular Biology, Johns Hopkins Bloomberg School of Public Health, Baltimore, Maryland, United States of America.
