# Supplementary material for: Aberrant gene expression by Sertoli cells in infertile men with Sertoli cell-only syndrome
Source: PLoS One. 2019 May 9;14(5):e0216586. doi: 10.1371/journal.pone.0216586 (PMC6508736; doi:10.1371/journal.pone.0216586)
Supplement: S1 Text — (DOCX) [file pone.0216586.s001.docx]

**Supplementary Methods**

**Defining signature transcripts of rat Sertoli cell**

The transcriptomes of adult rat Leydig cells, adult Sertoli cells, spermatogonia, pachytene spermatocytes and round spermatids were previously defined by use of rat genome 230 2.0 Affymetrix arrays [1, 2]. Those analyses identified 12786 Affymetrix probe sets as expressed by mature Rat Sertoli cells, which mapped to 4662 characterized genes [1].. Cell files for the analyses of all of these cell types were downloaded from the NCBI Geo database (GSE8978, GSE26703) and imported into Partek Genomics Suite (Partek Incorporated, St. Louis MO). Using this program, we identified probe sets whose expressions were significantly (FDR< 0.05) and at least 4-fold higher in Sertoli cells than in any of the other 4 cell types. Probe sets were mapped to the most current annotation of the rat genome and orthologues identified in the human testis transcriptome. This analysis identified 539 orthologues of rat Sertoli cell transcripts that were present in normal human testes, as defined by our RNAseq analysis. Given our focus on the spermatogonial stem cell niche, we then asked if the 539 transcripts ascribed to Sertoli cells included all of the transcripts in the Sertoli-Sertoli Cell Junction Dynamics pathway in: (<http://pathcards.genecards.org/>)

This resulted in our adding 26 transcripts to the original 539. We also asked if the list of putative Sertoli cell transcripts contained transcripts encoding all of the potential ligands for the cytokine and growth factor receptors that are transcriptionally expressed by human spermatogonial stem cells [3]. The potential ligands were determined by searching the String database of protein-protein interaction networks (<https://string-db.org/>) for ligands that bound each receptor. This resulted in our adding an additional 58 transcripts resulting in 623 transcripts that we subject to the secondary screen to identify human Sertoli cell signature transcripts.

**The secondary screen to identify signature transcripts of human Sertoli cells**

We used a secondary screen to complete our definition of human Sertoli cell signature transcripts. This screen was based on our RNAseq analysis of the transcriptome of human testes with normal spermatogenesis, published data on the transcriptomes of human Leydig cells [4], on human spermatogenic cells [5], and the relative numbers of spermatogenic cells, Sertoli cells and Leydig cells in a normal human testis [6, 7]. The human spermatogenic cells included in the published data, as well as abbreviations for each cell type and their relative numbers which are present in parentheses were: A dark spermatogonia (Adx; 11), A pale spermatogonia (Apx; 9.3), Leptotene/zygotene spermatocytes (L/Z; 12.2), early pachytene spermatocytes (eP; 12.2), late pachytene spermatocytes (lP; 14.4) and round spermatids (Round sptd; 45.3). The relative numbers of Leydig cells (4.2) and Sertoli cells (16.8) were also calculated from the published data. The sum of the relative numbers of cells in normal human testes was therefore, 125.2. Data for expression of each transcript in each sample were normalized to the amount of beta actin in that sample and these normalized data were used in the following equation to estimate the average beta-actin normalized expression of a transcript by Sertoli cells:

Relative expression per Sertoli cell = ((Testis x 125.2)-(Ad x 11)-(Ap x 9.3)-(L/Z x 12.2)-(eP x 12.2)-(lP x 14.2)-(round sptd x 45.3)-(Leydig x 4.2))/16.8.

Next, to estimate the sum of the expression of a transcript by all cells, we multiplied the level of beta-actin normalized expression by each cell type by the relative numbers of each cell type:

Sum of Expression by all cells= (Adx x 11)+(Apx x 9.3)+(L/Z x 12.2) +(eP x 12.2)+ (lP x 14.2)+(Round sptd x 45.3)+(Leydig x 4.2)+(Sertoli x 16.8).

Finally, to estimate the fraction of the total testis content of a given transcript that is attributed to Sertoli cells, we multiplied our estimate of the average expression of a transcript by Sertoli cells by 16.8 and then divided this product by the sum of expression by all cells:

Fraction of total testis content of a transcript that is attributed to Sertoli cells = (Sertoli x 16.8) / Sum of Expression by all cells.

In all but 1 case, if these calculations showed that Sertoli cells produced at minimum 60% of a given transcript and if Sertoli cells also expressed higher levels of that transcript than any of the other cell types, we defined that transcript as a signature transcript of human Sertoli cells. (See: Appendix Table S2). The one exception was CDH2 (N cadherin), which is a major Sertoli cell integral membrane cell adhesion protein. (Appendix Table S1 shows our estimate that Sertoli cells produced 46% of all CDH2 mRNA in a human testis; Appendix Table S2 shows that CDH2 expression does not differ between normal and SCO testes.) This secondary screen defined 243 transcripts as signature transcripts of human Sertoli cells.

**Quantitative RT-PCR analysis of FGF8 and beta actin mRNA**

**RNA Isolation.** Total RNA was isolated from fragments of testes obtained from three biopsies of cadaveric testes with normal spermatogenesis and from testis biopsies obtained from patients that were confirmed to have Sertoli Cell Only Syndrome (SCO). RNA was isolated using miRCURY RNA Isolation Kit (Cat No.300111, Exiqon Inc. Vadbeak, Denmark). First, the frozen tissue was completely homogenized in a lysis solution using TissueRuptor. The lysate was incubated with proteinase K for protein removal and loaded onto a spin column. Genomic DNA was removed directly on the column with DNase I (Qiagen) at final concentration of 0.25 Kunitz unit/microliter during the 20 minutes incubation. Eluted total RNA was checked for purity and integrity on an Agilent Bioanalyzer 2100 (Agilent Technologies, CA, USA). Only RNA’s with RNA Integrity number (RIN) greater or equal to 7 that did not show any RNA degradation were used. RNA concentration was measured with a fluorescence based quantitation assay using Q-bit fluorometer (Life Technology, NY, USA).

**Quantitative RT-PCR.** cDNA was synthesized with random hexamer primers from 0.1ug of total RNA using the Transcriptor First Strand cDNA Synthesis Kit (Roche Diagnostics). FGF8 mRNA levels were measured with the use of dualcolor multiplex qRT-PCR with the Universal Probe Library (UPL) hydrolysis probe set on a Light Cycler 480 instrument (Roche Diagnostics). UPL Assay Design Center (<https://lifescience.roche.com/en_us/brands/universal->probe-library.html#assay-design-center) was used to design the multiplex assay. FGF8 gene expression was determined with forward primer 5’-caggtcctggccaacaag -3’ and reverse primer 5’ -cttccaaaggtgtccgtctc -3’ using UPL Probe #53. For relative quantification target gene expression was normalized to β-actin (ACTB) using a proprietary Human ACTB Gene Assay (Roche Diagnostics). All qRT-PCR reactions were run in triplicate on 96-well plates at two different cDNA levels. The 20-uL reaction mixture contained 5 uL of either 1:5 or 4:5 diluted cDNA and 0.2uM UPL probe for either target gene or ACTB probe, 0.6uM forward and reverse primers for FGF8, 0.2uM forward and reverse primers for ACTB, and 1x LightCycler 480 Probes Master mix. All experiments were performed using a thermo-cycler (LightCycler 480, Roche) with the following settings: denaturation at 95°C for 10 min, 45 cycles of 95°C for 10 s and 60°C for 30 s, and a cooling cycle to 55°C with single fluorescence acquisitions at the end of each cycle. Target gene/ACTB expression ratios were determined with LightCycler 480 Relative Quantification software (Roche Diagnostics Corp.) using crossing points that were determined by the second derivate maximum or Fit Points methods after color compensation applied by the software. Statistical differences in target gene expression were considered statistically significant at p< 0.05.

**Flow Cytometric analysis of FGF8-expressing cells in normal and SCO human testes**

**Preparation of single cell suspensions of human testis and their labeling with antibodies to Sox9 and FGF8.** FACs analysis was performed as previously described [8]. We prepared single cell suspensions from small pieces of cadaveric testes with normal spermatogenesis or from biopsies of human SCO testes. Tissues were minced in 0.5 ml of ice-cold DMEM with 10% fetal bovine serum and loaded onto disposable disaggregator Medicon unit with 50 **μ**m separator mesh (BD Biosciences, San Jose, CA) filled with 0.5 ml of DMEM/10% FBS medium) processed in the BD Medimachine for 50 seconds and filtered through 50 **μ**m and then 30 **μ**m Filcon units. The single cell suspensions were washed with DMEM/10%FBS medium, centrifuged at 300 g for 5 min, and fixed in BD Cytofix fixation buffer for 20min. Fixed cells were washed twice with BD Perm/Wash buffer and again centrifuged at 500 g for 5 min. Following a 10 minute permeabilization with Perm/Wash solution cells were stained with fluorescently-labeled antibodies for FGF8 (Abcam ab89550) and for the Sertoli cell marker, SOX9. To determine the assay background for each preparation of cells, aliquots of cells that had not been incubated with antibodies were subjected to FACS analysis.

Anti-SOX9 was labeled with AF-448; anti-FGF8 was labeled with AF-647. Following a 1 h room temperature incubation of the cell suspension with labeled antibodies, the cells were washed twice with DMEM/10%FBS medium and suspended in 300 **μ**l of staining solution (BD Biosciences).

**FACS analysis***.* Unstained and stained cells were analyzed on a BD Accuri C6 flow cytometer with automatic plate loader and shaker. Data (fluorescence, forward and side scatter for each cell) were managed with BD C6 Software Version 1.0 and FlowJo v10.2. Each sample of cells was subjected to two separate FACS analyses and 100,000 cells were recorded per analysis. Cells were analyzed with a BD Accuri™ C6 flow cytometer equipped with two solid-state lasers: 488 and 640 nm. The following optical filters were

used: FL1 533/30 nm, FL2 585/40 nm, FL3 **>** 670 nm and FL4 675/25 nm.

Data from 100 000 cells were recorded for each flow cytometry run. Data that were gathered included the amount of fluorescence from bound antibodies and cell size, as measured by side scatter. Data were analyzed using BD C6 Software Version 1.0 and FlowJo v10.2.

**References**

1. Johnston DS, Wright WW, Dicandeloro P, Wilson E, Kopf GS, Jelinsky SA. Stage-specific gene expression is a fundamental characteristic of rat spermatogenic cells and Sertoli cells. Proc Natl Acad Sci U S A. 2008;105(24):8315-20. Epub 2008/06/12. doi: 10.1073/pnas.0709854105. PubMed PMID: 18544648; PubMed Central PMCID: PMC2448834.

2. Stanley EL, Johnston DS, Fan J, Papadopoulos V, Chen H, Ge RS, et al. Stem Leydig cell differentiation: gene expression during development of the adult rat population of Leydig cells. Biol Reprod. 2011;85(6):1161-6. Epub 2011/08/13. doi: 10.1095/biolreprod.111.091850. PubMed PMID: 21832170; PubMed Central PMCID: PMC3223250.

3. Guo J, Grow EJ, Yi C, Mlcochova H, Maher GJ, Lindskog C, et al. Chromatin and Single-Cell RNA-Seq Profiling Reveal Dynamic Signaling and Metabolic Transitions during Human Spermatogonial Stem Cell Development. Cell Stem Cell. 2017;21(4):533-46 e6. Epub 2017/10/07. doi: 10.1016/j.stem.2017.09.003. PubMed PMID: 28985528; PubMed Central PMCID: PMCPMC5832720.

4. Lottrup G, Belling K, Leffers H, Nielsen JE, Dalgaard MD, Juul A, et al. Comparison of global gene expression profiles of microdissected human foetal Leydig cells with their normal and hyperplastic adult equivalents. Mol Hum Reprod. 2017;23(5):339-54. Epub 2017/03/24. doi: 10.1093/molehr/gax012. PubMed PMID: 28333300.

5. Jan SZ, Vormer TL, Jongejan A, Roling MD, Silber SJ, de Rooij DG, et al. Unraveling transcriptome dynamics in human spermatogenesis. Development. 2017;144(20):3659-73. Epub 2017/09/25. doi: 10.1242/dev.152413. PubMed PMID: 28935708; PubMed Central PMCID: PMCPMC5675447.

6. Paniagua R, Codesal J, Nistal M, Rodriguez MC, Santamaria L. Quantification of cell types throughout the cycle of the human seminiferous epithelium and their DNA content. A new approach to the spermatogonial stem cell in man. Anat Embryol (Berl). 1987;176(2):225-30. Epub 1987/01/01. PubMed PMID: 3619074.

7. Petersen PM, Seieroe K, Pakkenberg B. The total number of Leydig and Sertoli cells in the testes of men across various age groups - a stereological study. J Anat. 2015;226(2):175-9. Epub 2014/12/30. doi: 10.1111/joa.12261. PubMed PMID: 25545958; PubMed Central PMCID: PMCPMC4304573.

8. Singh D, Paduch DA, Schlegel PN, Orwig KE, Mielnik A, Bolyakov A, et al. The production of glial cell line-derived neurotrophic factor by human sertoli cells is substantially reduced in sertoli cell-only testes. Hum Reprod. 2017;32(5):1108-17. Epub 2017/04/04. doi: 10.1093/humrep/dex061. PubMed PMID: 28369535.
