# Supplementary figures and images for: Aberrant gene expression by Sertoli cells in infertile men with Sertoli cell-only syndrome
Source: PLoS One. 2019 May 9;14(5):e0216586. doi: 10.1371/journal.pone.0216586 (PMC6508736; doi:10.1371/journal.pone.0216586)

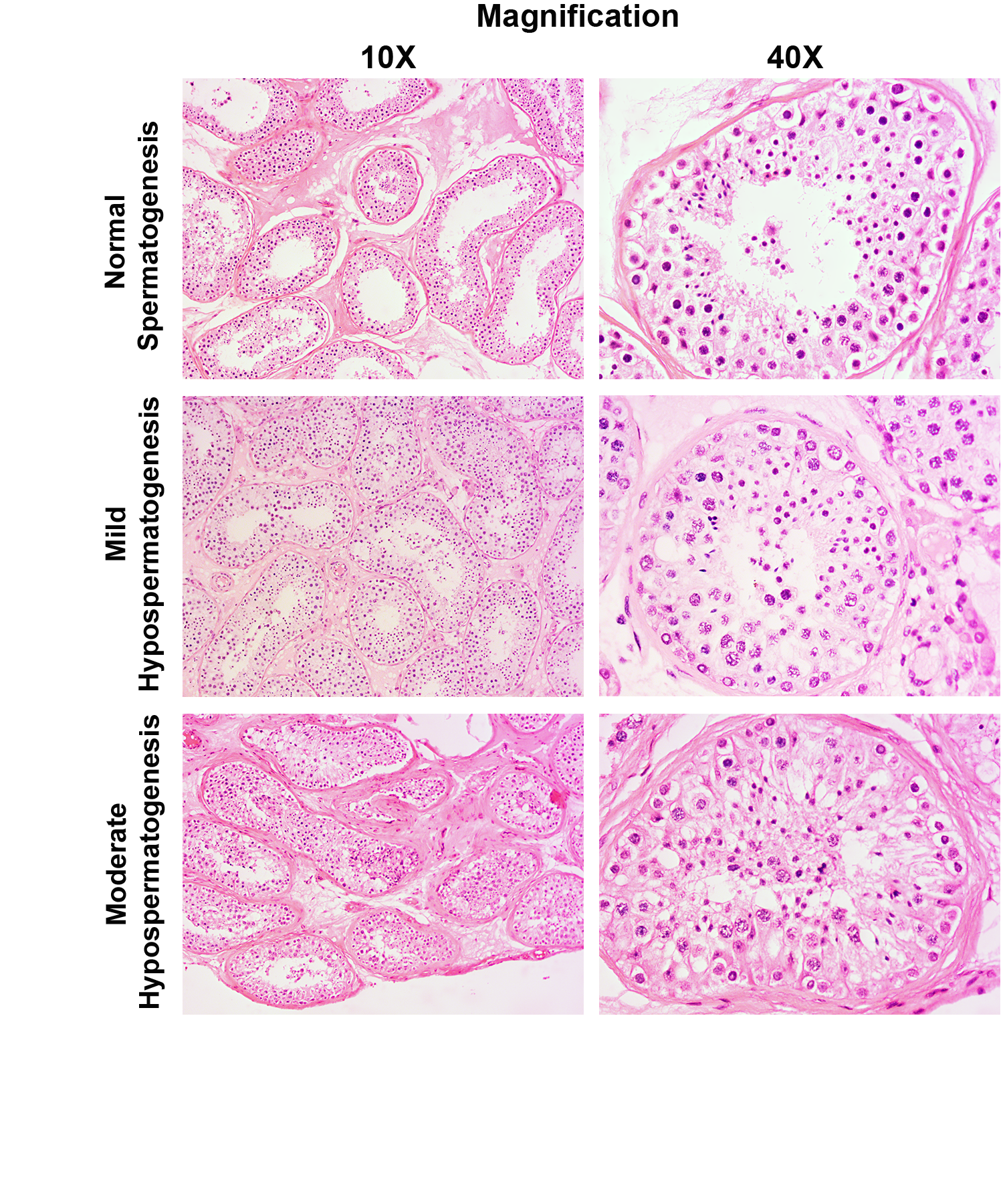

Supplement: S1 Fig — The testes with these three histologies are all referred to in the manuscript as exhibiting complete spermatogenesis. Photomicrographs were captured using 10X and 40x microscope lenses. (TIF) [file pone.0216586.s002.tif]

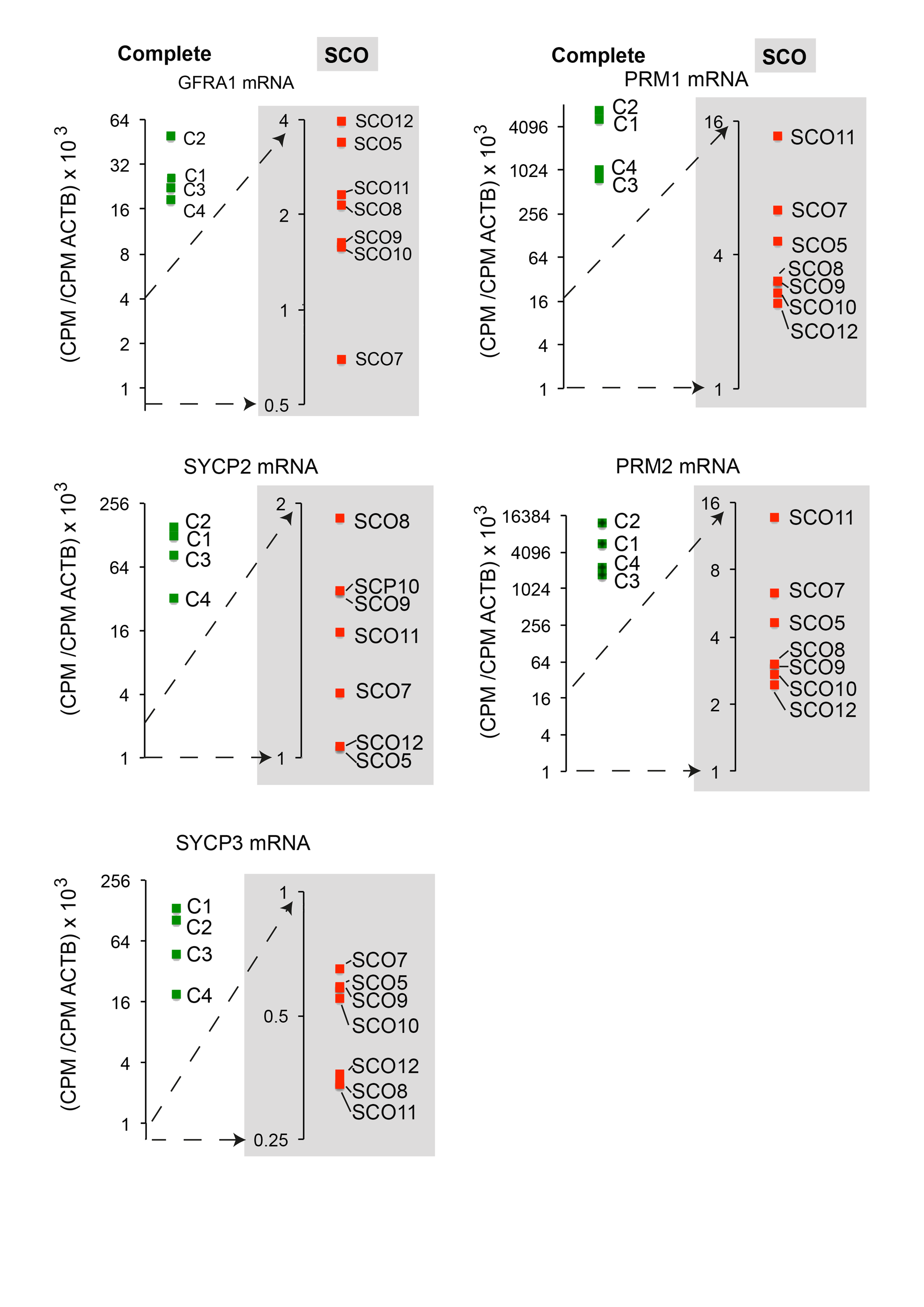

Supplement: S2 Fig — Data are from total testis transcriptomes and are expressed as CPM for each of the above transcripts divided by CPM for beta actin. (TIF) [file pone.0216586.s003.tif]

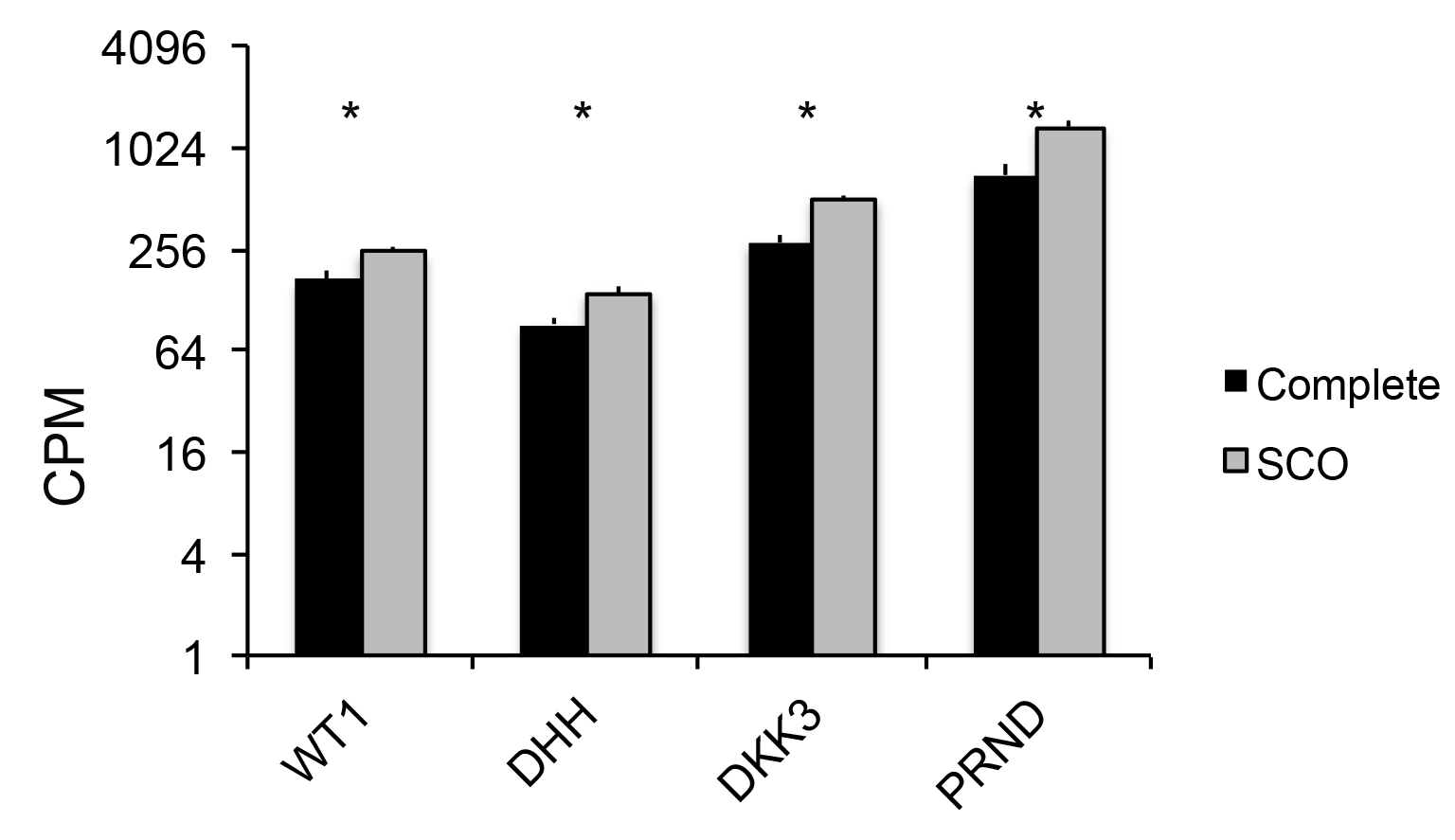

Supplement: S3 Fig — Data (mean+ SEM) are expressed CPM. An asterisk over a pair of bars indicates a significant difference between testes with complete spermatogenesis and SCO testes (FDR≤ 0.05). (TIF) [file pone.0216586.s004.tif]

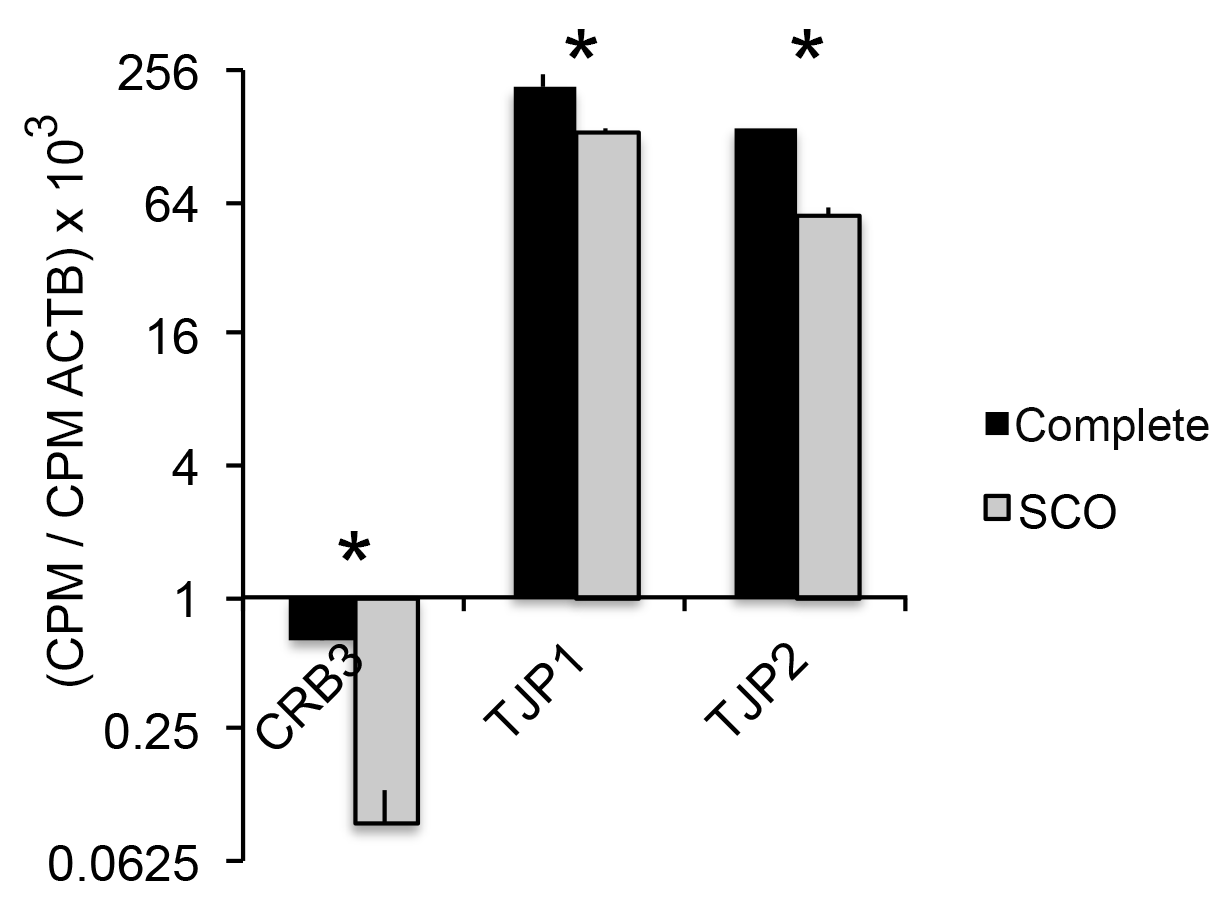

Supplement: S4 Fig — Data (mean + CPM) are expressed as CPM in the total testis transcriptome divided by CPM of ACTB in the same sample. Asterisks over a pair of bars indicate that normalized expression of a transcript differs between testes with complete spermatogenesis and SCO testes (p≤0.005). (TIF) [file pone.0216586.s005.tif]

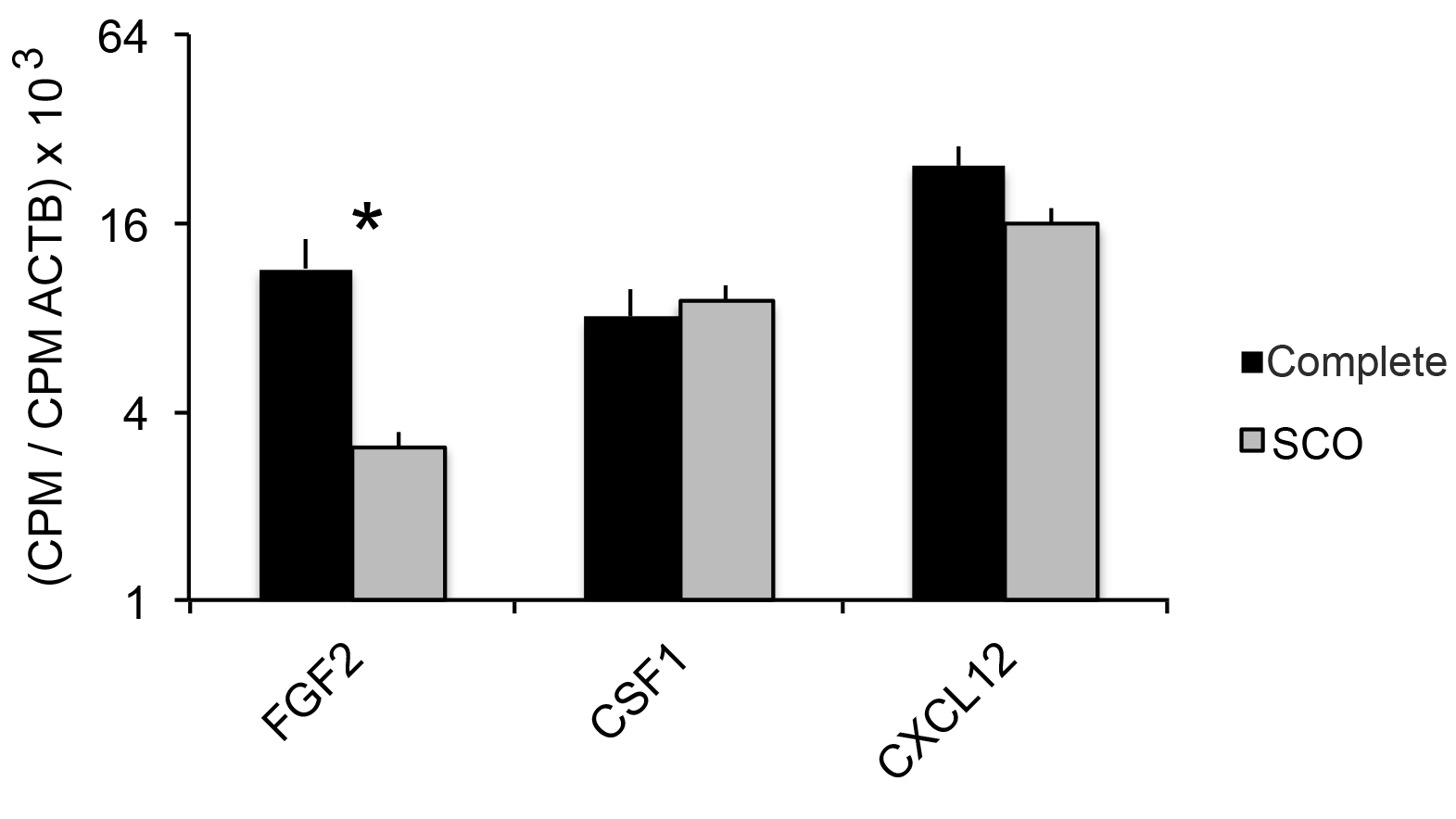

Supplement: S5 Fig — Data (mean + SEM) are expressed as CPM in the total testis transcriptome divided by CPM of ACTB in the same sample. An asterisk over a pair of bars indicates a significant difference between testes with complete spermatogenesis and SCO testes (p≤0.005). (TIF) [file pone.0216586.s006.tif]
